# Multi-Representation of Symbolic and Nonsymbolic Numerical Magnitude in Chinese Number Processing

**DOI:** 10.1371/journal.pone.0019373

**Published:** 2011-04-26

**Authors:** Chao Liu, Honghong Tang, Yue-Jia Luo, Xiaoqin Mai

**Affiliations:** 1 State Key Laboratory of Cognitive Neuroscience and Learning, Beijing Normal University, Beijing, China; 2 Center for Human Growth and Development, University of Michigan, Ann Arbor, Michigan, United States of America; 3 Key Laboratory of Mental Health, Institute of Psychology, Chinese Academy of Sciences, Beijing, China; Cuban Neuroscience Center, Cuba

## Abstract

Numerical information can be conveyed by either symbolic or nonsymbolic representation. Some symbolic numerals can also be identified as nonsymbolic quantities defined by the number of lines (e.g., I, II, III in Roman and 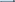, 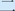, 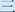 in Japanese Kanji and Chinese). Here we report that such multi-representation of magnitude can facilitate the processing of these numerals under certain circumstances. In a magnitude comparison task judging 1 to 9 (except 5) Chinese and Arabic numerals presented at the foveal (at the center) or parafoveal (3° left or right of the center) location, multi-representational small-value Chinese numerals showed a processing advantage over single-representational Arabic numerals and large-value Chinese numerals only in the parafoveal condition, demonstrated by lower error rates and faster reaction times. Further event-related potential (ERP) analysis showed that such a processing advantage was not reflected by traditional ERP components identified in previous studies of number processing, such as N1 or P2p. Instead, the difference was found much later in a N400 component between 300–550 msec over parietal regions, suggesting that those behavioral differences may not be due to early processing of visual identification, but later processing of subitizing or accessing mental number line when lacking attentional resources. These results suggest that there could be three stages of number processing represented separately by the N1, P2p and N400 ERP components. In addition, numerical information can be represented simultaneously by both symbolic and nonsymbolic systems, which will facilitate number processing in certain situations.

## Introduction

Number concepts can be represented in two forms. One is as a concrete nonsymbolic quantity, such as a set of dots or lines, the other is an abstract symbolic numeral, such as Arabic numerals. In some languages, symbolic numerals consist of concrete quantities, for example, in Roman, the numerals for one, two, and three are represented by vertical lines (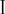, 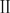, 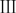). Chinese and Japanese (Kanji) also use this type of representation for small numerals, except that they use horizontal lines (one *yi1/ichi* 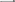, two *er4/ni* 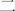, and three *san1/san* 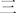). Do these types of multi-representational numerals have advantages over single-representational abstract symbolic numerals (e.g., Arabic numerals) under certain circumstances? More specifically, could properties that adhere to a concrete quantity facilitate the processing of these multi-representational numerals to some extent?

Different theories have different interpretations of whether and how numerical information could be represented by multiple systems. According to McCloskey's abstract modular model [1], [2], numerical information is encoded by either an Arabic or a verbal representation, which will converge onto a common abstract internal representation during arithmetic operation and mental calculation. Similarly, Dehaene's [3] triple-code model suggests that numerical information can be represented as a visual Arabic numeral form, an auditory verbal word frame, or an analogical form serves as a common abstract representation of magnitude information, or internal number line. Moreover, in this triple-code model, Arabic, verbal and analogical magnitude can directly communicate with each other through an asemantic transcoding route. As a contrast to these models with a common abstract representation, Campbell and Clark [4], [5] proposed an encoding-complex model that denies the existence of a single, abstract number representation. Instead, they suggested that there is an interactive network of specialized notation-specific codes, including both visual and phonological representations. Recently, based upon neuroimaging studies that revealed notation-dependent activation in the brain, Cohen Kadosh and his colleagues supported this notation-specific representation by suggesting that numbers in different notations can be represented separately in the human parietal lobe, and thus challenged the abstract representation of numerical information in number processing [6], [7], [8], [9].

As far as multi-representational numerals such as small-value Roman and Chinese numerals were concerned, they might have two possible advantages. First, according to the triple-code model of number presentation [3], [10], in a magnitude comparison task, the Arabic input is transformed into an analogue magnitude code before the comparison can be performed in the internal analogical number line [11]. Nonetheless, this transformation processing might not be necessary for multi-representational numerals. Being concrete quantities already, these numerals could directly provide magnitude information in the analogical number line and thus facilitate the magnitude judgment. Similar prediction could also be drawn from notation-dependent theories [4], [5], [7], such that multi-representational numerals, as concrete quantities, can directly access magnitude information embedded in their particular internal representations.

Second, multi-representational numerals might allow for subitizing, which means a small number of concrete quantity, such as 1–4 dots, can be processed more rapidly, accurately, and confidently than a larger number of concrete quantity, such as 6–9 dots, which requires effortful counting. Such differences are reflected by the slightly increasing reaction times and error rates for enumerating the first three or four items but rapidly increasing after four [12], [13], [14]. As a concrete quantity, the Roman and Chinese numerals for one, two and three could potentially evoke subitizing processing as several separate lines and thus facilitate the access of magnitude information in these numerals.

However, these possible facilitations have seldom been reported in previous studies with Roman, Chinese and Japanese Kanji numerals. Compared with Arabic numerals, Chinese numerals have been found to have faster responses in a naming task but slower responses in a magnitude comparison task [15]. Chinese or Japanese Kanji numerals have been found to show no advantage over Arabic numerals in the numerical memory span task [16] and the parity judgment task [17]. Event-related potential (ERP) and functional magnetic resonance imaging (fMRI) studies also found that Chinese or Japanese Kanji numerals showed similar behavioral responses and ERP components P1 and N1 as Arabic numerals in a magnitude comparison task [18], and elicited a more significant activation than Arabic numerals only in the left inferior occipital and fusiform gyrus without any parietal activation in a naming task [19]. In one exception, Ito and Hatta [20] found that Janpanese Kanji and Arabic numerals showed similar overall reaction times but different congruity effects in an implicit physical size judgment task. Nevertheless, they only used five numeral pairs (3, 4, 7, 8, and 9) in their study, which might not be a representative sample of Japanese Kanji and Arabic numerals.

Nonetheless, none of those studies focused on multi-representational small-value Chinese numerals and manipulated variables that could strengthen the difference, such as attention. The idea that attention may influence number processing derives from neuroimaging studies. A region called the posterior superior parietal lobe (PSPL) is found to be active in both number processing tasks [21], [22], [23], [24], [25], [26], [27], [28] and attention related tasks [29], [30], [31]. Dehaene and his colleagues therefore proposed that this region of the brain, in addition to being involved in attention orienting in space, can also contribute to attentional selection on other mental dimensions that are analogous to space, such as numbers [32]. Specifically, according to their triple-code model, they suggested that the process of shifting attention to select locations in space can also be engaged when accessing specific quantities on the internal mental number line. In addition, such number-based attention would be particularly necessary in tasks that require the selection of one amongst several quantities [32], e.g., the magnitude comparison task in which participants are required to decide which of two numerals or quantities is the larger one [33], [34], [35]. Originating from neuroimaging studies, this proposal was directly supported by a behavioral study that showed that merely looking at Arabic numerals caused a shift in covert attention to the left or right side, depending upon the numerals' magnitudes [36].

Therefore, if the putative influence of attention on number processing is true, such that spatial attention is necessary for orienting and accessing the magnitude information in the internal mental number line, we could expect that multi-representational numerals will rely less on spatial attention than single-representational numerals because they can bypass the transformation-to-abstract representation processing and thus require less attentional resources. As a result, the facilitation of concrete quantity representation in multi-representational numerals might be observed behaviorally when amplified by the lack of global attentional resources.

Similarly, research has also found that both subtizing and counting highly depend on attention [37], [38], [39], [40]. According to the spatial indexing hypothesis, our visual system is capable of indexing a limited number (∼4) of objects (or feature clusters) after parallel preattentive grouping processes [41], so the attentional demands in subitizing might only originate from selecting and binding the preattentively processed features [42], [43], which could be highly parallel and automatic, thus requiring minimal attentional resources. However, a number of recent literatures suggest that subitizing can be also influenced by manipulations of attentive load thus is not pre-attentive. For example, subitizing is highly compromised during attention blink ([38], [44], [45]. Other studies have shown that subitizing suffers from dual tasks, when spatial attention is diverted from the estimation task [38], [46], [47], [48], [49]. No matter subitizing is pre-attentive or not, when lacking attentional resources, multi-representational numerals might evoke dissimilar subitizing processing than single-representational numerals and thus demonstrate observable difference than single-representational numerals.

Exploring the corresponding neural correlates could provide even more enlightenment. In previous ERP studies using either single-representational abstract numerals such as Arabic or English verbalization, or in other studies with concrete quantities such as a set of dots, two ERP components have been repeatedly found to be associated with number processing: a early negative component N1 peaking around 100–150 msec and a late posterior positivity P2p peaking around 250 msec [50], [51], [52]. The subitizing processing has been found to be associated with the N1 component reflecting the distribution of spatial attention [51], [52], whereas the P2p component reflects later number processing of representation, estimation and comparison [34], [50], [51]. Therefore, if we find the advantage of multi-representational numerals being reflected by early ERP components such as N1, then it could be attributed to the early subitizing processing; however, if we find the advantage being represented by other later ERP components such as P2p, then it could be attributed to the later processing of accessing and operating with numerical magnitude in the analogical number line.

Several different paradigms have been established to modulate attention, e.g., dual tasks [38], [46], [47], [48], [49], the Ponser task[53], or foveal/parafoveal presentation[54]. In our previous studies investigating Chinese number processing[55], [56], we used the paradigm developed by Enns and DiLollo [54], in which stimuli were presented randomly at the foveal and parafoveal location, and found comparable behavioral results as the well-established Posner task [53] in visual attention studies [57] (see Materials S1). In the current study, we will use this paradigm again to explore the possible behavioral and ERP differences between multi-representational small Chinese numerals and single-representational Arabic numerals, as well as their underlying mechanism. We predict that participants' performances could be better for those multi-representational small Chinese numerals than single-representational Arabic numerals in the parafoveal condition. Brain-wave differences between multi-representational and single-representational numerals could further reveal the underlying cognitive mechanism.

## Materials and Methods

### Participants

Nineteen healthy right-handed native Chinese-speaking undergraduates (10 males, *M* age  =  19.55 years) participated the study for payment. The recruitment of participants was approved by IRBs at the Institute of Psychology, Chinese Academy of Sciences. All participants' written informed consents have been obtained.

### Stimulus and apparatus

**Figure 1:**
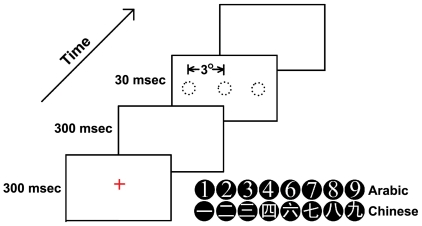
Experimental procedure: (a) A red cross (0.4°visual angle) appeared as a fixation point in the center of the screen for 300 msec; (b) A blank screen appeared for 300 msec; (c) One of the sixteen numerals appeared randomly in one of three locations of two conditions (foveal: center; parafoveal: 3°left or right to the center, with 60, 30 and 30 presentation times, respectively) for 30 msec; (d) A blank screen appeared until participants responded. There was a 500 msec interval before the next trial. The task was to judge whether each presented Chinese or Arabic numeral was smaller or larger than the number five using two index fingers.

The stimuli were white numerals (subtended 0.6°×0.6°) in black disks (diameter 0.7°) (Fig. 1). Arabic (in Times New Roman font) and Chinese (in Heiti font) numerals from 1 to 9 (excluding 5) were used. All stimuli were presented against a white background on a PC display (Refresh Rate 100 Hz, resolution 600×800, programmed with E-PRIME1.1). Participants viewed the stimuli from a distance of about 47 cm.

### Procedure and task

We adopted the paradigm developed by Enns and DiLollo [54] and used in our previous studies[55], [56], in which stimuli were presented randomly at the foveal and parafoveal location (see Fig. 1 for the experimental procedure). The participants' task was to decide whether each presented Chinese or Arabic numeral (from 1 to 9, except 5) was larger or smaller than 5 by pressing one of two separate keys on the keyboard (“F” and “J”) with their left and right index fingers. The key assignment was counterbalanced between participants. Both accuracy and speed were emphasized in the instructions. Participants were encouraged to maintain focus on the center of the screen starting from the presentation of the fixation cross until response execution.

Each numeral was presented 30 times at the left side, 30 times at the right side, and 60 times at the center. The entire experiment, comprised of 168 practice trials and 1920 formal experimental trials, was divided into 12 experimental blocks separated by participant-controlled rest, and took about 60 minutes in total.

### ERP recording

The electroencephalogram (EEG) was recorded from 64 scalp sites using tin electrodes mounted in an elastic cap (NeuroScan Inc.), with the reference on the linked left and right mastoids. The vertical electrooculagram (EOG) was recorded with electrodes placed above and below the left eye. All inter-electrode impedance was maintained below 5 kΩ. The EEG and EOG were amplified using a 0.05–100 Hz bandpass and continuously sampled at 500 Hz/channel for off-line analysis. Trials with EOG artifacts (mean EOG voltage exceeding ±100 µV) and those contaminated with artifacts due to amplifier clipping, bursts of electromyographic (EMG) activity, or peak-to-peak deflection exceeding ±100 µV were excluded from averaging. A 3Dspace FASTRAK digitizer was used to record the 3D coordinates of each electrode and of three fiducial landmarks (the left and right preauricular points and the nasion).

### ERP data analysis and statistics

**Figure 2:**
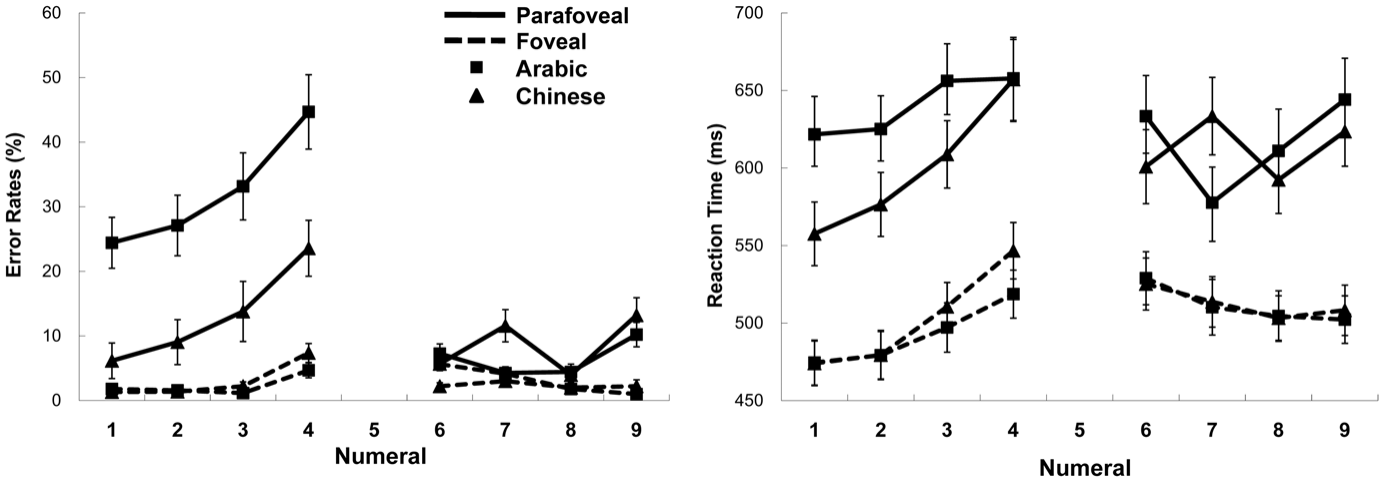
Error rates and reaction times data for Chinese and Arabic numerals (error bars show 1 SE). Significant differences between small-value Chinese and Arabic numerals were found for both error rates (*p*<.001) and reaction times (*p*<.001) data only in the parafoveal condition.

The Arabic numeral 4 received extremely high error rates (44%) in the parafoveal condition (Fig. 2), probably due to its visual similarity to the Chinese large-value numeral ten 十, with only an additional slash at the upper left corner, which could severely disrupt the participant when those numerals were presented so fast at the parafoveal location. As a result, both the pair of numerals four (4 and 四) and six (6 and 六) were omitted in further ERP analysis to keep the trial number balanced between small-value and large-value numerals, even though six showed no noticeable difference than seven, eight and nine. EEGs for all numerals (except four and six) in the foveal and parafoveal conditions were then analyzed with a 2 by 2 design (Magnitude: Small (1–3) *vs.* Large (7–9); Notation: Arabic *vs.* Chinese), resulting in four types of defined conditions: (Small Chinese, Large Chinese, Small Arabic and Large Arabic).

**Figure 3:**
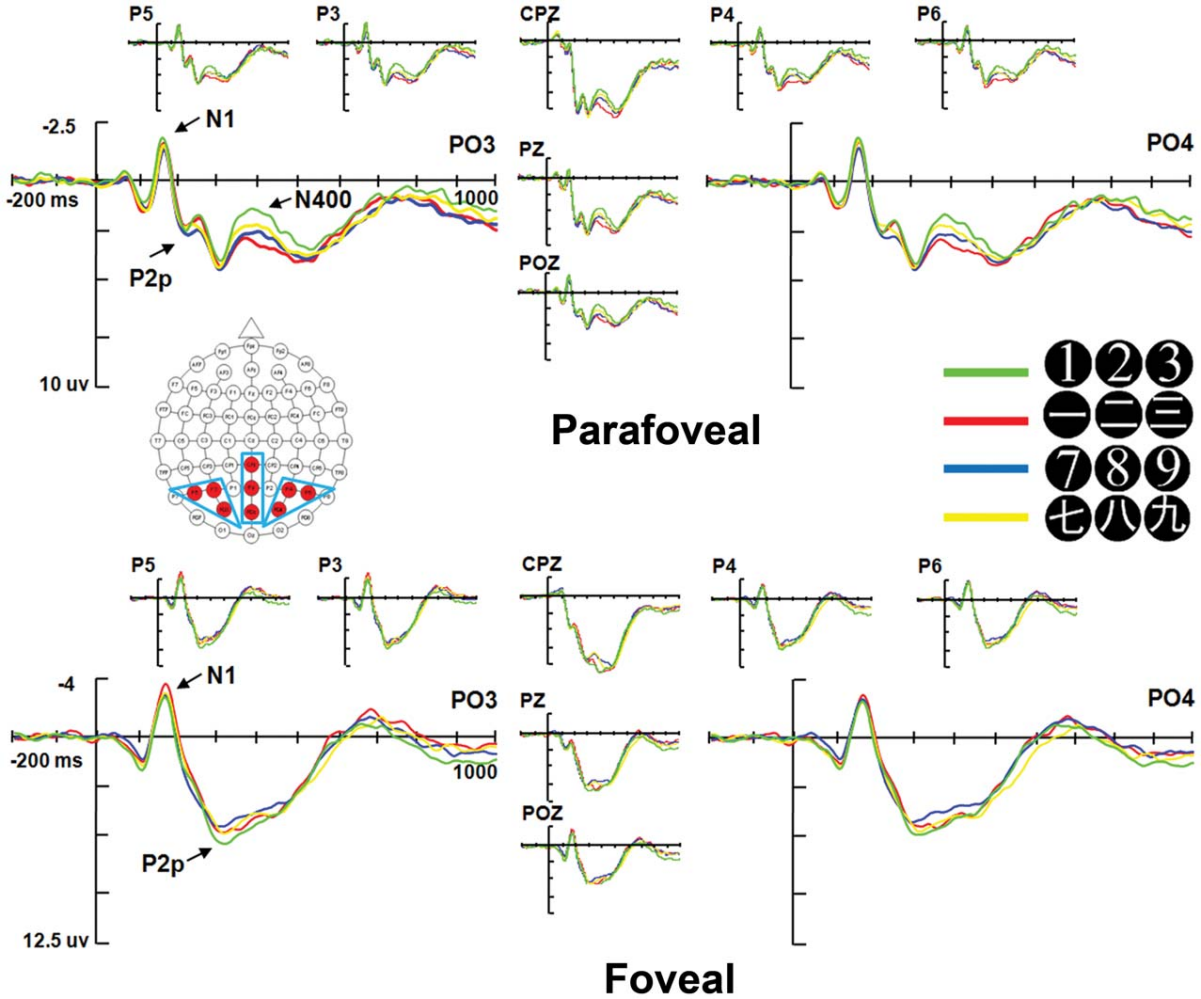
Grand average ERPs at nine posterior electrode sites (red dots in the electrode layout diagram). Both small and large-value Chinese and Arabic numerals in the parafoveal (top) and foveal (bottom) condition were shown. PO3 and PO4 were enlarged to represent the parieto-occipito-temporal regions, focused-on in previous number processing studies [50], [51], [52], [62]. Electrodes were grouped into three sites in the analysis: left, middle and right (blue boxes in the electrode layout diagram). Differences between small Chinese and small Arabic numerals can be found primarily in a widely distributed N400 component (*p*<.05) in the parafoveal condition. No significant differences between Chinese and Arabic numerals in the N1 and P2p components were found in the foveal condition.

The ERP waveforms were time-locked to the onset of the numeral stimuli. Averaged epochs begin with a 200 msec pre-stimuli baseline and last for 1000 msec. Based on previous studies of numerical cognition [34], [58], we at first focused our analyses on two well-established ERP components, N1 (negative peak voltages between 100–200 msec) and P2p (positive peak voltages between 200–320 msec), over three posterior electrode groups (Left sites: P3, P5, PO3; Middle sites: CPz, Pz, POz; Right sites: P4, P6, PO4)[51], [52]. However, after visually inspecting the grand average waveform in the foveal and parafoveal conditions (Fig. 3), we found that the only apparent distinction between small Chinese and Arabic numerals in the parafoveal condition was in a widely spread N400 component, which was not found in the foveal condition (Fig. 3). This N400 component has not been reported by any previous number processing studies. We added this component in our analyses (N400: mean voltages between 300–550 msec.

The baseline-to-peak amplitude and latencies of N1 and P2p and mean amplitude of N400 were then analyzed using repeated measures analysis of variance (ANOVAs) with Location (Foveal *vs.* Parafoveal) by Magnitude (Small *vs.* Large) by Notation (Arabic *vs.* Chinese) by Sites (Left *vs.* Middle *vs.* Right). The *p* values of all main effects and interactions were corrected using the Greenhouse-Geisser method for repeated-measures effects.

### ERP current density distribution

The averaged current density distribution of the difference wave (small-value Chinese minus small-value Arabic) in the time range of 300–550 msec (Peak  =  402 msec) was reconstructed using the Minimum Norm method with Curry V4.5 (Neurosoft, Inc.). The mean values of these individual 3D coordinates of each electrode and of three fiducial landmarks (the left and right preauricular points and the nasion) were calculated over the 19 participants and were fed into Curry. A computer algorithm was automatically performed in Curry to calculate the best-fit sphere encompassed by the array of electrode sites and to determine their spherical coordinates. The spherical coordinates for each site averaged across all participants were then used for the ERP current density analysis.

### Dipole source analysis

The Brain Electrical Source Analysis program (BESA, version 5.1.6, MEGIS Software GmbH, Germany) was used to perform dipole source analysis with the four-shell ellipsoidal head model. The same difference wave (small Chinese minus small Arabic) as the current density distribution analysis was examined. Principal component analysis (PCA) was employed in the interval of 390–414 msec on the peak of the N400 component in order to estimate the number of dipoles needed to explain the difference wave. When the number of dipoles was determined with PCA, software automatically determined the dipole location. The relevant residual variance criterion was used to evaluate whether this model explained the data best and accounted for most of the variance.

## Results

### Behavioral results

Outliers were minimized by excluding trials with a response time >1200 msec or <200 msec; 2.3% of all responses [55], [57]. Mean error rates and correct reaction times (RTs) for Chinese and Arabic numerals in the foveal and parafoveal conditions were plotted (Fig. 2).

A 2 (Location: Foveal *vs.* Parafoveal) by 2 (Magnitude: Small 1–4 *vs.* Large 6–9) by 2 (Notation: Arabic *vs.* Chinese) repeated measures ANOVA on both error rates and RTs data revealed significant main effects of Location (Error rates: *F* (1, 18) = 50.05, *p*<.001; RTs: *F* (1, 18) = 147.96, *p*<.001), Magnitude (Error rates: *F* (1, 18) = 16.42, *p*<.001), and Notation (Error rates: *F* (1, 18) = 18.52, *p*<.001; RTs: *F* (1, 18) = 6.95, *p*<.05), as well as significant interactions for Location by Notation (Error rates: *F* (1, 18) = 30.65, *p*<.001; RTs: *F* (1, 18) = 30.70, *p*<.001), Location by Magnitude (Error rates: *F* (1, 18) = 17.98, *p*<.001), Notation by Magnitude (Error rates: *F* (1, 18) = 21.78, *p*<.001) and Location by Notation by Magnitude (Error rates: *F* (1, 18) = 28.96, *p*<.001; RTs: *F* (1, 18) = 10.40, *p*<.01). These results suggest that large and small Chinese and Arabic numerals do differ when being presented in the foveal and parafoveal condition.

A further Magnitude (Small *vs.* Large) by Notation (Arabic *vs.* Chinese) repeated measures ANOVA on both error rates and RTs data was then conducted for both foveal and parafoveal conditions. No significant main effects or interaction was found in the foveal condition. However, significant main effect of Notation and Magnitude by Notation interaction were found for both error rates and RTs data in the parafoveal condition (Error rates: Notation, *F* (1, 18) = 23.76, *p*<.001, Magnitude by Notation, *F* (1, 18) = 26.18, *p*<.001; RTs: Notation, *F* (1, 18) = 20.22, *p*<.001, Magnitude by Notation, *F* (1, 18) = 7.44, *p*<.05). Post-hoc analyses with Bonferroni corrections showed significant Notation main effect for small numerals, Error rates, *p*<.001; RTs: *p*<.001. As shown in Fig. 2, participants made fewer errors and responded faster for small Chinese numerals (Error rates: *Mean*  =  13.26%; RTs: *Mean*  =  600.01 msec) than small Arabic numerals (Error rates: *Mean*  =  32.23%; RTs: *Mean*  =  640.22 msec) when they were presented at the parafoveal location, especially for the numerals one, two and three. Nonetheless, such differences were not found between large Chinese and Arabic numerals, nor when small numerals were presented at the foveal location. This pattern of Chinese vs. Arabic numerals processing in the foveal and parafoveal conditions has been repeatedly found in previous behavioral studies using similar paradigms [55], [57]. In these studies, Arabic numeral 1 and Chinese numeral 一 are almost visually identical, except that one is a vertical line and the other is a horizontal line. However, participants' performance differed greatly for these two visually and semantically similar numerals (Fig. 2), further indicated that it is the common properties of small Chinese numerals as a whole (e.g., being multi-representational numerals), rather than the individual characteristic of each numeral per se, that contributes to their processing advantage over small Arabic numerals.

### ERP results

#### N1

An omnibus 2 (Location: Foveal *vs.* Parafoveal) by 2 (Magnitude: 1–3 *vs.* 7–9) by 2 (Notation: Arabic vs. Chinese) by 3 (Sites: Left *vs.* Middle *vs.* Right) repeated measures ANOVA on both amplitude and latency data was conducted first. Results showed a significant main effect of Location in both amplitude and latency data (amplitude: *F* (1, 18) = 25.98, *p*<.001; latency: *F* (1, 18) = 23.21, *p*<.001, indicating that Location indeed has an influence on the amplitude and latency of the N1 component. A Magnitude by Notation by Sites repeated measures ANOVA on both amplitude and latency data was then conducted for both foveal and parafoveal conditions.

#### Foveal condition

The results of amplitude data revealed a significant main effect of Sites, *F*(2, 36) = 7.83, *p*<.01, Magnitude by Sites interaction, *F*(2,36) = 3.86, *p*<.05, and Notation by Sites interaction, *F*(2,36) = 4.01, *p*<.05. The amplitude of N1 was smaller at the middle (*M* = −1.83 µV) than the left (*M* = −3.93 µV) and right (*M* = −3.60 µV) sites. Post-hoc analyses with Bonferroni corrections showed no significant Notation or Magnitude main effect at all sites. No main effects or interactions were found for latency data except the Sites, *F*(2, 36) = 37.68, *p*<.001. The latency of N1 was faster at the middle (*M* = 133.23 msec) than the left (*M* = 59.19 msec) and right (*M* = 156.67 msec) sites.

#### Parafoveal condition

The results of amplitude data revealed a significant main effect of Sites, *F*(2, 36) = 4.02, *p*<.05. The amplitude of N1 was smaller at the middle (*M* = −1.90 µV) than the left (*M* = −2.63 µV) and right (*M* = −2.64 µV) sites. Post-hoc analyses with Bonferroni corrections showed no significant Notation effect at all sites, but a significant Magnitude effect for Arabic numerals at all three sites (Left, *p*<.05; Middle, *p*<.05; Right, *p*<.05), such that small Arabic numerals elicited larger N1 (*M* = −3.00, −2.27, −2.95 µV, respectively) than large Arabic numerals (*M* = −2.33, −1.64, −2.38 µV, respectively). No main effects or interactions were found for latency data except the Sites, *F*(2, 36) = 9.08, *p*<.01. The latency of N1 was faster at the middle (*M* = 146.02 msec) than the left (*M* = 158.47 msec) and right (*M* = 158.58 msec) sites.

#### P2p

Similar omnibus 2 (Location: Foveal *vs.* Parafoveal) by 2 (Magnitude: 1–3 *vs.* 7–9) by 2 (Notation: Arabic vs. Chinese) by 3 (Sites: Left *vs.* Middle *vs.* Right) repeated measures ANOVA on both amplitude and latency data was conducted first. Results showed a significant main effect of Attention and Magnitude in both amplitude and latency data (amplitude: *F* (1, 18) = 12.01, *p*<.001, *F* (1, 18) = 6.78, *p*<.01; latency: *F* (1, 18) = 8.84, *p*<.005, *F* (1, 18) = 7.67, *p*<.05, indicating that attention indeed has an influence on the amplitude and latency of the P2p component. A Magnitude by Notation by Sites repeated measures ANOVA on both amplitude and latency data was then conducted for both foveal and parafoveal conditions.

#### Foveal condition

The results of amplitude data revealed only a significant main effect of Sites, *F*(2, 36) = 23.64, *p*<.001. The amplitude of P2p was larger at the middle (*M* = 8.07 µV) than the left (*M* = 5.49 µV) and right (*M* = 6.04 µV) sites. Post-hoc analyses with Bonferroni corrections showed no significant Notation or Magnitude effect at all sites. No main effects or interactions were found for latency data.

#### Parafoveal condition

The results of amplitude data revealed only a significant main effect of Sites, *F*(2, 36) = 26.99, *p*<.001. The amplitude of P2p was larger at the middle (*M* = 7.23 µV) than the left (*M* = 4.46 µV) and right (*M* = 4.67 µV) sites. Post-hoc analyses with Bonferroni corrections showed no significant Notation or Magnitude effects at all sites. No main effects or interactions were found for latency data except the Sites, *F*(2, 36) = 4.95, *p*<.05. The latency of P2p was slower at the right (*M* = 253.07 msec) than the left (*M*  = 244.00 msec) and middle (*M*  = 245.17 msec) sites.

In summary, Arabic and Chinese numerals, no matter small or large, demonstrated no significant differences in both the N1 and P2p component. The only reliable difference found in N1 and P2p components was that the middle posterior site yielded a more positive going waveform than the left and right posterior sites, reflected by smaller N1 and large P2p, which was consistent in both foveal and parafoveal conditions.

#### N400

Similar omnibus 2 (Location: Foveal *vs.* Parafoveal) by 2 (Magnitude: 1–3 *vs.* 7–9) by 2 (Notation: Arabic vs. Chinese) by 3 (Sites: Left *vs.* Middle *vs.* Right) repeated measures ANOVA on mean amplitude of N400 was conducted first. Results showed a significant main effect of Attention, Notation and Magnitude on N400 amplitude (*F* (1, 18) = 20.78, *p*<.001, *F* (1, 18) = 8.74, *p*<.001, *F* (1, 18) = 5.97, *p*<.05, respectively), indicating that location indeed has an influence on amplitude of the N400 component. A Magnitude by Notation by Sites repeated measures ANOVA on amplitude data was then conducted for both foveal and parafoveal conditions.

#### Foveal condition

The results of amplitude data revealed only a significant main effect of Sites, *F*(2, 36) = 14.56, *p*<.001. The amplitude of N400 was larger at the middle (*M* = 5.65 µV) than the left (*M* = 4.01 µV) and right (*M* = 3.76 µV) sites. Post-hoc analyses with Bonferroni corrections showed no significant Notation or Magnitude effect at all sites.

#### Parafoveal condition

**Figure 4:**
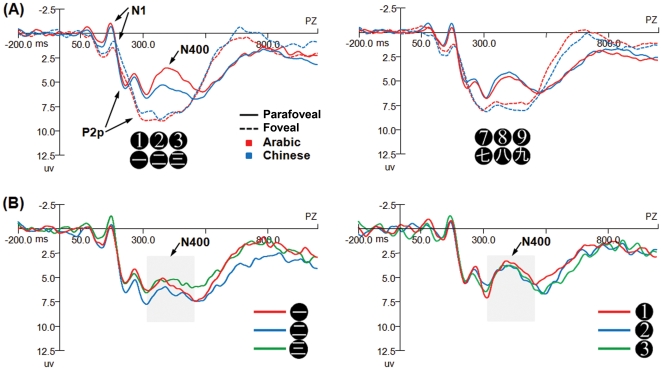
**(A) Grand average ERPs at Pz.** Small numerals, one, two, and three, in which Chinese showed smaller N400 amplitude than Arabic (*p*<.05), are on the left side. The large numerals, seven, eight, and nine, in which Chinese and Arabic showed no difference, are on the right side as a contrast. Here no significant differences between Chinese and Arabic numerals in the N1 and P2p components were found for both foveal and parafoveal conditions. **(B)** **Grand average ERPs elicited by the small numerals in the parafoveal condition.** Chinese numerals one, two and three all elicited an attenuated N400 component compared with their Arabic counterparts, *p*<.05, *p*<.05, *p* = .09, respectively, indicating that the overall N400 difference between small Chinese and Arabic numerals when combined together is not due to a quirk of any single numeral.

Similar repeated measures ANOVA of Magnitude (Large vs. Small) by Notation (Arabic *vs.* Chinese) by Sites (Left *vs.* Middle *vs.* Right) on N400 amplitudes revealed significant main effect of Notation, *F* (1, 18) = 7.14, *p*<.05, and Sites, *F*(2, 36) = 14.71, *p*<.001, indicating that the N400 amplitude was smaller for Chinese (*M* = 4.64 µV) than Arabic (*M* = 4.20 µV) numerals, and was smaller at the middle (*M* = 5.44 µV) than the left (*M* = 3.84 µV) and right (*M* = 3.97 µV) sites. There was also a Magnitude by Notation interaction, *F* (1, 18) = 5.42, *p*<.05. Small Chinese numerals showed smaller N400 (*M* = 4.92 µV) than that of Arabic small numerals (*M* = 3.85 µV); whereas large Chinese (*M* = 4.36 µV) and Arabic (*M* = 4.54 µV) numerals did not show such a difference (Fig. 4 A). Post-hoc analyses with Bonferroni corrections showed significant N400 differences between small Chinese and small Arabic at all three sites (Left, *p*<.05; Middle, *p*<.05; Right, *p*<.01), such that small Chinese numerals (*M* = 4.27, 6.00, 4.28 µV, respectively) elicited smaller N400 than small Arabic numerals (*M* = 3.29, 4.84, 3.42 µV, respectively). In addition, the post-hoc analyses revealed significant or marginal significant N400 differences between small Chinese and large Chinese at all three sites (Left, *p* = .10; Middle, *p*<.05; Right, *p* = .07), such that small Chinese numerals (*M* = 4.27, 6.00, 4.28 µV, respectively) elicited smaller N400 than large Chinese numerals (*M* = 3.83, 5.34, 3.90 µV, respectively). These results showed that multi-representational small Chinese numerals showed significantly smaller N400 than single-representational small Arabic and large Chinese numerals. To further explore the differences between small Chinese and small Arabic numerals in the parafoveal condition, we also conducted a Numeral (one *vs.* two *vs.* three) by Notation (Arabic *vs.* Chinese) by Sites (Left *vs.* Middle *vs.* Right) repeated measures ANOVA on N400 amplitude, which showed significant main effects of Notation, *F* (1, 18) = 8.05, *p*<.05 and Sites, *F*(2, 36) = 14.68, *p*<.001, such that the N400 amplitude was smaller for Chinese (*M* = 4.91 µV) than Arabic (*M* = 3.79 µV) numerals, was smaller at the middle (*M* = 5.38 µV) than the left (*M* = 3.76 µV) and right (*M* = 3.92 µV) sites. (Fig. 4 B). Post-hoc analyses with Bonferroni corrections showed that significant or marginal significant N400 differences between Chinese and Arabic numerals one, two and three could be found at all three sites, Left: *p* = .07, *p*<.01, *p* = .10; Middle: *p*<.05, *p*<.05, *p* = .09; Right: *p*<.05, *p*<.05, *p* = .07; respectively. Such notation effect for small numerals one, two and three was not found for either the N1 or the P2p component (Fig. 4B).

**Figure 5:**
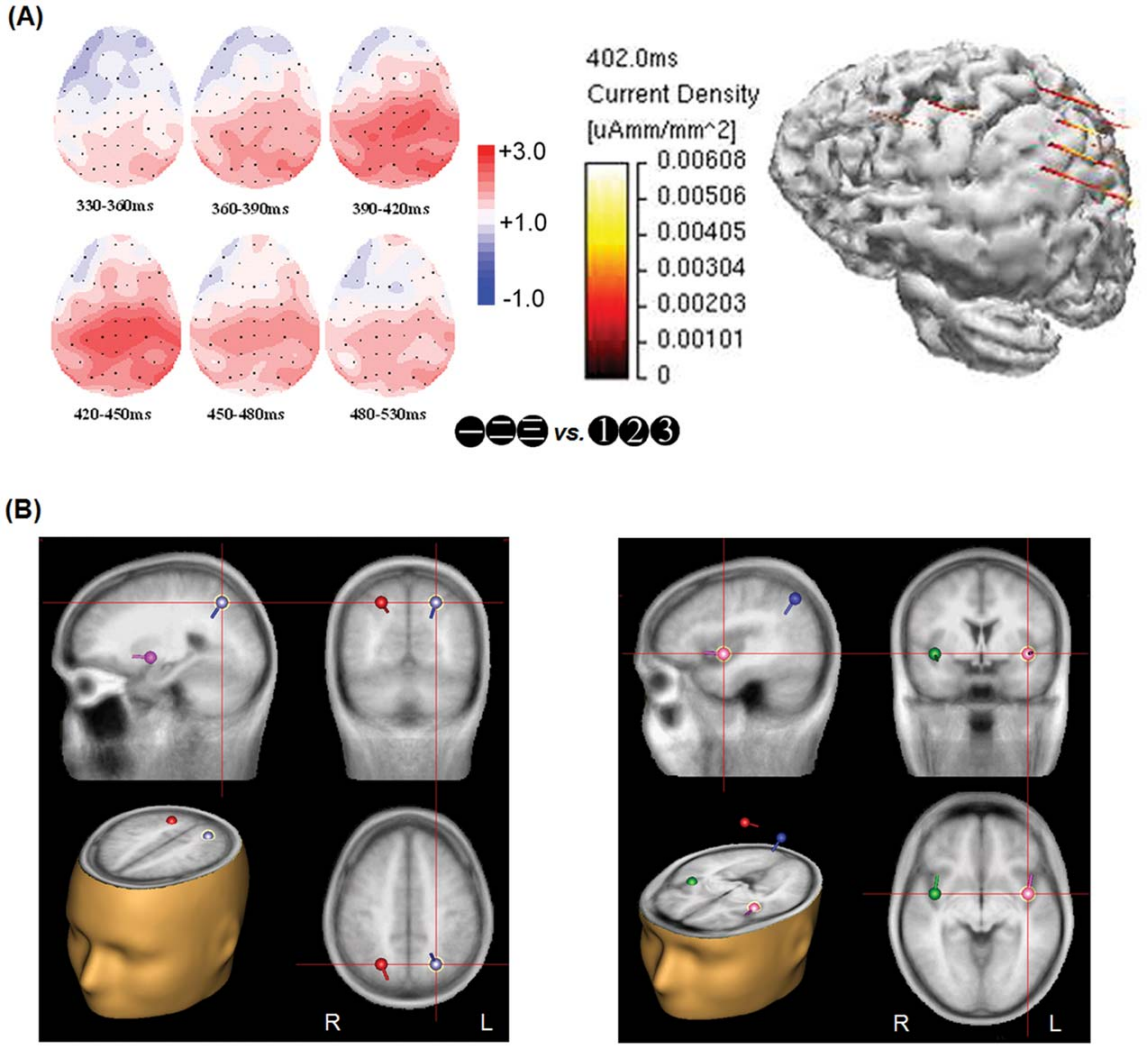
**(A) The voltage maps of the difference wave (small-value Chinese - small-value Arabic) in the parafoveal condition.** The results showed strong activity in the central-parietal region between 390–450 msec (Left). The averaged current density distribution of the same difference wave at 402 msec was reconstructed with the Minimum Norm method in Curry V4.5 (Neurosoft, Inc.), which also demonstrated a strong current density in the parietal cortex (Right). (**B**) **Dipole source analysis of the same difference wave.** The results revealed two bilaterally symmetric dipole pairs that can account for most of the variance in the data, residual variance (R.V.)  = 6.57%, with one pair in the bilateral superior parietal lobe, Brodmann area 7, Talairach coordinates *x* = ±23, *y* = −62, *z* = 44 (left), and the other in the bilateral insula, Brodmann area 13, *x* = ±40, *y* = −1, *z* = −4 (right).

Further analyses with the voltage map of difference waves (small Chinese minus small Arabic) showed strong activity at the parietal regions. The averaged current density distribution of the same difference waves during the time range of 300–550 ms, also demonstrated a current density primarily in the parietal cortex (Fig. 5 A).

### Source localization

PCA indicated that two principal components together could explain 98.9% of the variance in the data in the 390–414 msec time window. Therefore, based upon previous findings that revealed bilateral parietal activation for number processing [32], [34], [59], [60], as well as the fact that we did not find significant N400 differences between the left and right sites, two bilaterally symmetric dipole pairs were fitted in the model with no other restrictions as to the direction or location of the dipole. The results indicated that two dipole pairs located approximately in the bilateral superior parietal lobe, Brodmann area 7, Talairach coordinates *x* = ±23, *y* = −62, *z* = 44, and the bilateral insula, Brodmann area 13, *x* = ±40, *y* = −1, *z* = −4, explained the data best and accounted for most of the variance with a residual variance (R.V.) of 6.57% (Fig. 5B). Other solutions (e.g., one bilaterally symmetric dipole pair, two free dipoles or one symmetric pair with one free dipole) all resulted in R.V. larger than 10%.

### Correlation between behavioral and ERP results

**Figure 6:**
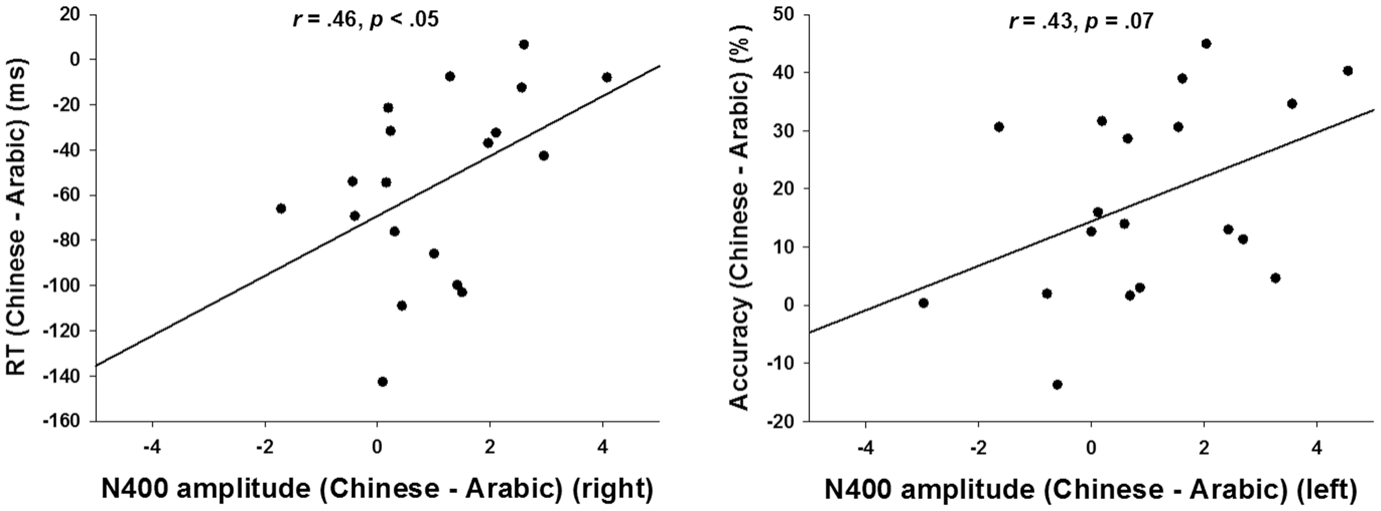
Correlation between the behavioral results and the ERP results. The difference between small Chinese and small Arabic in the amplitude of N400 component at the right site was positively correlated with the reaction time difference between them (left), whereas the difference between small Chinese and small Arabic in the amplitude of N400 component at the left site was positively correlated with the accuracy difference (right). These results indicate that the processing advantage of multi-representational small Chinese numerals is associated with the N400 component in the brain.

In order to further confirm that the behavioral differences we found between small Chinese and Arabic in the parafoveal condition are associated with the differences of the N400 ERP component, we also conducted correlation analyses correlating the accuracy and reaction time differences between small Chinese and small Arabic numerals with their N400 amplitude differences in the parafoveal condition at all three sites. The results revealed one significant and one marginally significant correlation, such that the difference between small Chinese and small Arabic in the amplitude of the N400 component at the right site was positively correlated with the reaction time difference between them, Pearson *r* = .46, *p*<.05, whereas the difference between small Chinese and small Arabic in the amplitude of the N400 component at the left site was positively correlated with the accuracy difference, Pearson *r* = .43, *p* = .07 (Fig. 6). These results indicate that the processing advantage of multi-representational small Chinese numerals is indeed associated with the N400 component in the brain.

## Discussion

Our study revealed that multi-representational numerals such as small-value Chinese numerals (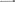, 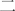, 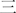) do have advantages over other single-representational abstract numerals such as small-value Arabic numerals (1, 2, 3) when being presented at the parafoveal location, reflected by lower error rates and faster reaction times, as well as a smaller amplitude of the N400 ERP component distributed over the parietal regions. In addition, numerical information can be represented simultaneously by both symbolic and nonsymbolic systems, which will facilitate number processing in certain situations.

In most previous magnitude comparison experiments, the performance of the verbal number words (e.g., English) is always worse than the written Arabic numerals. Nonetheless, it may be explained by the cost of perceiving the letters of those alphabetic number words [1]. In our results, Chinese and Arabic numerals showed no difference in behavioral results when being presented at the foveal location, which is in line with previous studies of Chinese [15], [17], [18] and Japanese Kanji number processing [16], [19], [61], indicating that Chinese numerals are as easy to be identified as Arabic numerals [15], [18]. However, remarkable differences were found when small Chinese and Arabic numerals were represented in the parafoveal condition. Participants made fewer errors and responded faster for small Chinese numerals than small Arabic numerals, especially for the numerals one, two and three (Fig. 2).

These behavioral results replicated our previous findings that, when presented at the parafoveal location, Chinese and Arabic numerals demonstrate different number processing effects depending on the magnitude [55], [57]. Only the small numerals one, two and three showed significant number notation effects (the performance of small Chinese numerals were better than small Arabic numerals) in the parafoveal location, even when participants were guided by endogenous attention cues (e.g., a red arrow in the center points to the left or right position) or exogenous attention cues (e.g., a red circle flashes at the left or right position) in a Posner task [57]. However, in a parity judgment task that did not require magnitude information, small Chinese numerals yielded no differences compared to small Arabic numerals in the parafoveal location, regardless of whether there were attention cues [56] (see Materials S1), which further indicates that the differences we observed in our studies are related to representing and accessing numerical magnitude rather than visual identification.

One possible interpretation of these differences is that small Chinese numerals evoked subitizing processing as small concrete quantities when lacking attentional resources. Nonetheless, our behavioral results questioned this hypothesis. Behaviorally, a subitizing account would predict shallower slope in error rates or RTs data for small numerals in the subitizing range than for large numerals in the counting range [12], [13], [14], yet the behavioral results in our study did not show such effects in both error rates and RTs for small Chinese numerals (Fig. 2), which indicates that the differences between small Chinese and other numerals might not be simply attributed to subitizing processing. However, we should note that we used a magnitude comparison task, so there will be a distance effect between 5 and the other number, which might interact with the subitizing processing and attenuated the effect behaviorally. Thus the behavioral results cannot rule out the subitizing account and it is still one possible explanation to the results.

The ERP data provide more information. Subitizing has been found to be reflected by early ERP component N1 [51], [52] and right temporo-parietal junction activation [62]; whereas in our results, it is not N1 but a much later superior parietal N400 component that was associated with the behavioral differences between small Chinese numerals and small Arabic numerals (Fig. 3, Fig. 4, Fig. 6), which has never been reported in previous number processing studies and could provide more insights on the time line of number representation and number processing.

### N400 and its role in number processing

As a well-established ERP component in language studies, N400, which occurs approximately 400 milliseconds after the stimulus onset, has been found to be related to a wide range of tasks that require semantic access, especially semantic conflict [63], [64], [65]. Studies have found that the N400 component is evoked by semantically inappropriate words (such as “John slept on the *socks*” when compared with “John slept on the *bed*”) [63], leading researchers to suggest that this component is an index of semantic access [66]. Additional research has shown that N400 amplitude is inversely related to the semantic congruence between a target word and its preceding context, even when the target word is not semantically anomalous [64]. Thus, the N400 is thought to be related not just to semantic access at a broad level, but specifically to how integral the meaning of a word or symbol to the semantic lexicon [67]. However, throughout previous number processing studies, accessing the “number semantics” seems to never evoke the N400 component, probably because the decimal number lexicon with only 10 numbers is much simpler than the semantic lexicon for a natural language. Instead, previous number processing studies have identified an earlier P2p component that corresponds to the access of number magnitude information [50], [51], [52].

Here, the presence of a N400 component following the P2p component during number processing in the parafoveal condition thus provides us with a special chance to revisit the time line of number processing established in previous studies [50], [51], [52]. That is, previously identified P2p components might not be detailed enough to represent the transaction and mapping processing in the numerical magnitude number line. A subsequent sub-processing of accessing the analogical magnitude that greatly depends on spatial attention could be separated and identified by a later N400 component, which is when multi-representational small Chinese numerals can apply their “short cut” of being a concrete magnitude, ready to facilitate the magnitude judgment, as can be reflected by an attenuated N400 component.

The voltage map and current density distribution of this N400 difference in the parietal region and dipole source in the bilateral superior parietal lobe (Fig. 5) further suggest that its origin could be similar to previously identified number processing areas in the parietal lobe, such as the posterior superior parietal lobe (PSPL) or the intraparietal sulcus (IPS) [32], [34], [62], [68], [69], [70], which have also been found in studies with Chinese and Japanese Kanji numerals [19], [71]. One variation is the dipole source in the bilateral insula, an area that has been found constantly involved in language processing, especially speech [72], [73], [74], [75], as well as number processing with Arabic numerals [33], [76]. It can perhaps explain the relatively more anterior spatial distribution of N400 in our results than the typical spatial distribution of N1 and P2p found in previous ERP studies of number processing [34], [50], [51], [52]. However, from the ERP data alone, it is hard to make any reliable judgment on the exact brain areas that are associated with the N400 found here. Further neuroimaging studies with precise spatial resolution are necessary to explore this question.

### Notation-dependent vs. notation-independent number representation

Our data also provides new evidence to the notation-dependent *vs.* notation-independent debate on number representation [6], [7], [8], [9]. On the one hand, the results that multi-representational small Chinese numerals can be processed differently from other single-representational Arabic and Chinese numerals under certain circumstances (not in the early processing of visual identification, but in the later processing of subitizing or accessing magnitude information in the internal representation) seem to directly support the idea that different notations can be represented differently in our mind without a common abstract representation. On the other hand, the fact that large Chinese numerals did not exhibit such an advantage indicates that variances of number representation can be found not only between different notations, but also within a particular notation, which to the best of our knowledge, has not been reported by any previous number processing studies. Although such variation of representations within a notation might be unique for multi-representational numerals such as small Chinese and Roman, it still suggests that a common abstract representation could be a more efficient solution as far as the cognitive resources are still limited in our mind, which otherwise could be ultimately overwhelmed by the total number of representations required for processing different numerals in different notations.

The key question here is, what cognitive process have the multi-representational numerals bypassed during the procedure of accessing magnitude information? The notation-independent theory could suggest that it is a transformation-to-abstract representation processing that has been bypassed, whereas the notation-depend theory could argue that such an abstract representation is not necessary and the multi-representational numerals simply access magnitude information from its concrete quantity representation, which is faster and easier than those abstract symbols which represents magnitude information in either the visual or semantic form. Both theories could allow a late processing stage of accessing the magnitude information, represented by the N400 component. Therefore, it is hard to tell from the current results alone which one is more proper. However, the present results indeed shed light on this debate because it proves that number processing effects could be amplified by manipulating the attentional resources involved, which can serve as a powerful tool to revisit those “null results” in previous studies [7], [9] and identify possible notation effects that have not been found using traditional paradigms.

### The influence of attention on number processing

Finally, the results directly support the existence of the influence of attention on number processing. Most previous number processing studies did not directly manipulate the attentional resources involved in the task, although numerous studies have shown that spatial attention is closely related to mapping numerical magnitude on the analogical number line [for a review, see 32], and the inverse influence from number to attention has been found in many studies, e.g., purely perceiving Arabic numerals can alter spatial attention according to the number magnitude [36]. In the present study, by intentionally manipulating attentional resources involved in the task, a notation-specific effect that could not be identified in those previous studies was enlarged and revealed in the parafoveal condition. These results thus support the idea that spatial attention is necessary in mapping and orienting magnitude information in an internal number line [32], thus the lacking attentional resources will impact the accessing and operating with numerical information to some extent. Such impairment has been found not only in the notation effect, but also other number processing effects such as the spatial-numerical association of response codes (SNARC) effect. In a study using the same paradigm as the present one, as well as the Posner task with either endogenous or exogenous attentional cues, we found that the SNARC effect was constantly attenuated for the large numerals (8 and 9) in the parafoveal condition of all three tasks, especially for Chinese numerals and in the task with exogenous cues [56]. As an important index of the left-to-right mental number line, the SNARC effect has been found to be rather consistent across different notations [77], [78], [79]. Its impairment in the parafoveal condition for large numerals, both Arabic and Chinese, therefore strongly supports the role of spatial attention in the orienting and mapping on the internal number line.

One interesting question is whether such an influence of attention on number processing is particularly strong for Chinese numerals. A study conducted by researcher in Taiwan has found that Chinese speakers can obtain two separate SNARC effects for Arabic or Chinese numerals, a horizontal one for the former and a vertical one for the latter, probably due to the fact that Chinese reading habit in Taiwan is mainly from top to bottom (e.g., in over 60% of printed books)[17]. Similar vertical SNARC effect has also been found for Japanese speakers with Arabic numerals [80]. Hence, it is possible that Chinese speakers can still rely on the top-to-bottom spatial attention for Chinese numerals when the left-to-right spatial attention is controlled in our previous studies, resulting in a less salient impairment of performance than Arabic numerals in the parafoveal condition. However, all participants in our studies are Chinese speakers from the mainland, where the reading habit has been mostly from left-to-right for decades. In fact, as far as we know, such a vertical SNARC effect has not yet been replicated with Chinese speakers on the mainland. Thus, it is less likely that such a top-to-bottom spatial attention found in Chinese speakers in Taiwan can account for all of our findings.

### Limitations

One possible alternative interpretation for the current results is familiarity and expertise, two variables that could contribute to many notation-specific findings, especially in developmental studies [81]. It could be argued that Chinese participants are more familiar with Chinese numerals than Arabic numerals because the former is generally used in writing as words whereas the latter is exclusively used in arithmetic and calculation as digits. As a result, they could receive different performance in different attention conditions. However, this account cannot explain the presence of the within-notation variation between small and large Chinese numerals, as well as the disappearance of such a variation in a parity judgment task [56], unless one can provide evidence that Chinese speakers are more familiar with small Chinese numerals and better at accessing their magnitudes than any other numerals, which to the best of our knowledge, does not exist in previous studies with Chinese and Japanese Kanji numerals.

In addition, an interesting finding in our behavioral results is that small numerals generally showed more errors than large numerals when being presented at the parafoveal condition (Fig. 2), which has been consistently found in our previous studies using different paradigms (e.g., the Posner task) [55], [56], [57]. Such an effect could not be predicted by any previous theories of number processing. Further studies investigating number processing at the parafoveal location are highly needed to explore this effect.

Of great interest, next, is whether comparable results could be observed in small Roman numerals (I, II, III) that have similar representations (but vertical) as Chinese small numerals. Unlike Roman, Chinese and Japanese Kanji characters are logographic scripts consisting of strokes (e.g., 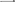, 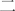, and 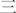 contains one, two and three strokes, respectively), which have been used to count numeral information by the Chinese ever since those characters were invented thousands of years ago. For example, the Chinese character *zheng4* 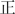, which “means main, correct, or positive”, has been widely used by Chinese and Japanese, even those illiterate, to count numerical information as multiples and remainders of five with its five vertical and horizontal strokes (e.g., four 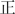 together means the value of twenty in analogous fashion to tally marks in the West). We could assume that the Chinese and Japanese have a long history of using multi-representational symbols and characters in their everyday lives. Thus the multi-presentation of symbolic and nonsymbolic numerical information might be a unique feature of Chinese number processing.

## Supporting Information

Materials S1 Supplementary Materials
(DOC)
