# Supplementary material for: Multi-Representation of Symbolic and Nonsymbolic Numerical Magnitude in Chinese Number Processing
Source: PLoS One. 2011 Apr 26;6(4):e19373. doi: 10.1371/journal.pone.0019373 (PMC3082580; doi:10.1371/journal.pone.0019373)
Supplement: Materials S1 — Supplementary Materials (DOC) [file pone.0019373.s001.doc]

Here is a summary of previous results in Chinese number processing under different attention conditions. All data shown here has been published in these three references:

1. **Liu, C.**, Mai, X. Q., & Fu, X. L. (2005). The influence of endogenous and exogenous attention on number processing (in Chinese). *Acta Psychologica Sinica*, *37*, 167-177.

Abstract (in English):

<http://www.fed.cuhk.edu.hk/en/aps/200500370002/0167.htm>

The pdf version (in Chinese):

[http://www-personal.umich.edu/~liuchao/files/The%20influence%20of%20endogenous%20and%20exogenous%20attention%20on%20number%20processing.pdf](http://www-personal.umich.edu/~liuchao/files/The influence of endogenous and exogenous attention on number processing.pdf)

1. **Liu, C.**, Mai, X. Q., & Fu, X. L. (2004b). The Spatial Numerical Association of Response Codes effect of number processing in different attention conditions (in Chinese). *Acta Psychologica Sinica*, *36*, 671-680.

Abstract (in English):

<http://www.fed.cuhk.edu.hk/en/aps/200400360006/0671.htm>

The pdf version (in Chinese):

[http://www-personal.umich.edu/~liuchao/files/The%20spatial%20numerical%20association%20of%20response%20codes%20effect%20of%20number%20processing%20in%20different%20attention%20conditions.pdf](http://www-personal.umich.edu/~liuchao/files/The spatial numerical association of response codes effect of number processing in different attention conditions.pdf)

1. **Liu**, **C.**, & Fu, X. L. (2004a). The influence of attention on the effects of number magnitude in number comparison task (in Chinese). *Acta Psychologica Sinica*, *36*, 307-314.

Abstract (in English):

<http://www.fed.cuhk.edu.hk/en/aps/200400360003/0307.htm>

The pdf version (in Chinese):

[http://www-personal.umich.edu/~liuchao/files/The%20influence%20of%20attention%20on%20the%20effects%20of%20number%20magnitude%20in%20number%20comparison%20task.pdf](http://www-personal.umich.edu/~liuchao/files/The influence of attention on the effects of number magnitude in number comparison task.pdf)

**1. Study one: Number comparison in different attention conditions without cue**


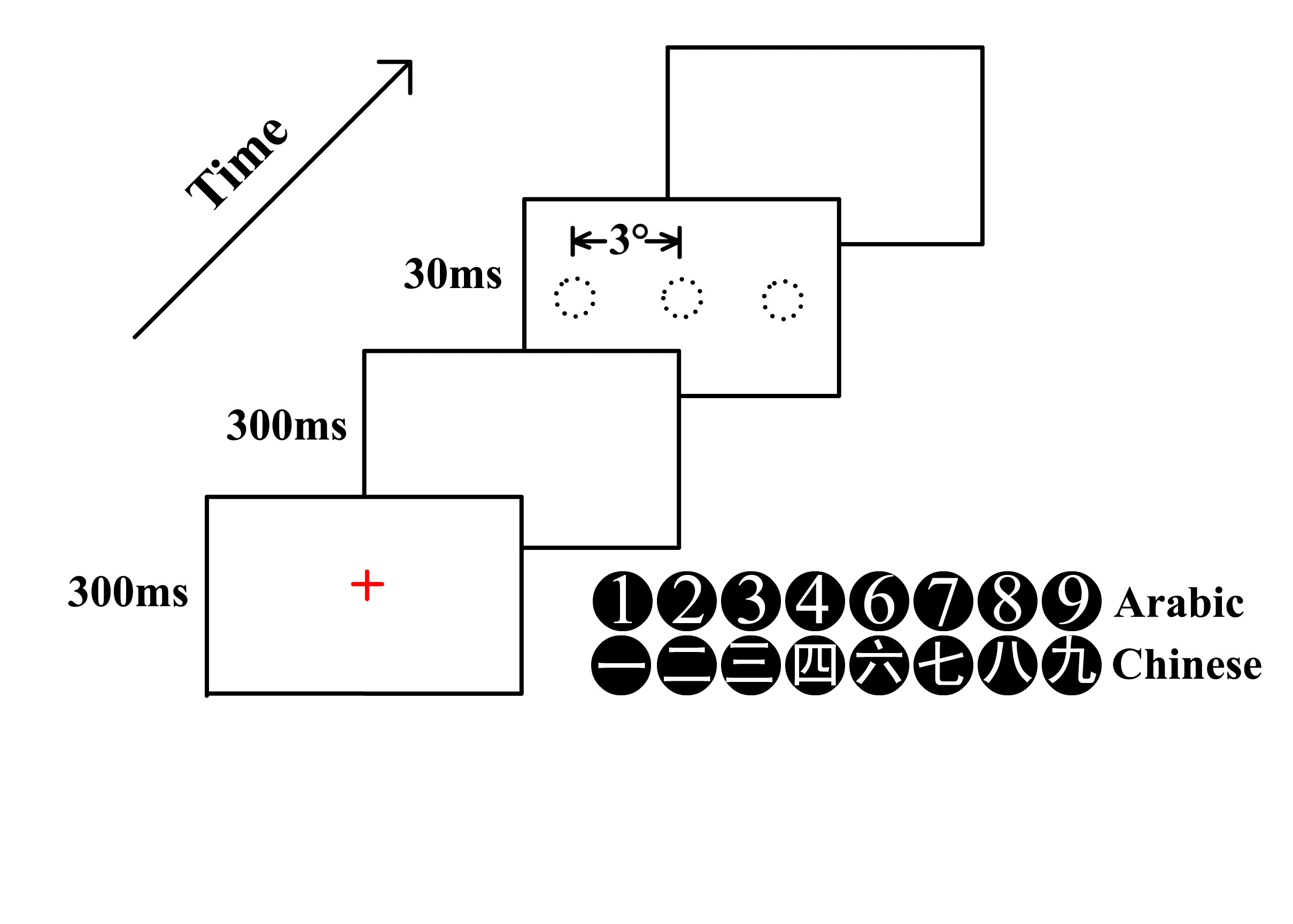


**Fig.1** Procedure (a) A red cross (0.4°visual angle) appeared as a fixation point in the center of the screen for 300 ms; (b) A blank screen appeared for 300 ms; (c) One of the sixteen numerals (subtended 0.6°× 0.6°on a black disk) appeared randomly in one of three locations of two conditions (attended: center, unattended: 3°left or right to the center, with 60, 30 and 30 presentation times, respectively) for 30 ms; (d) A blank screen appeared until participants responded.

1.1) Magnitude comparison (to 5) without cue


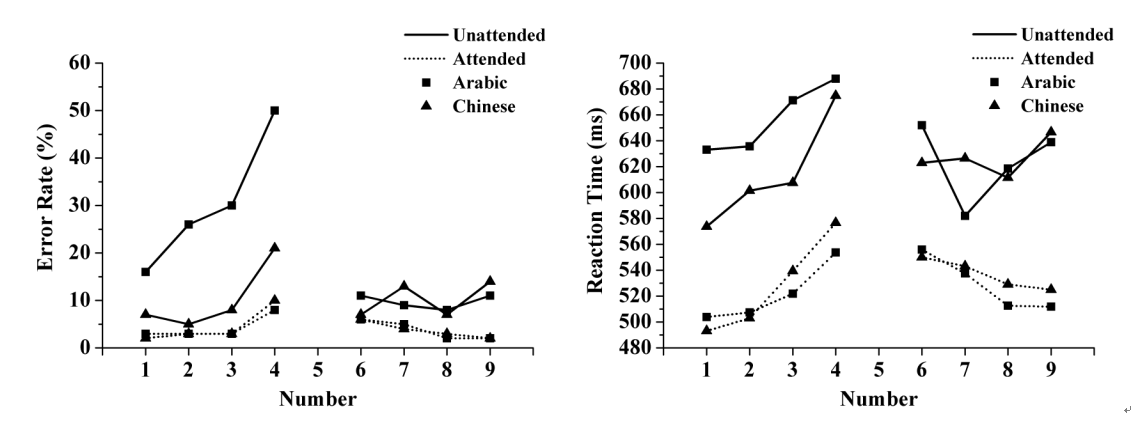


**Fig.2** Error rates of Magnitude comparison without cue

**Fig.3** RTs of Magnitude comparison without cue.

**
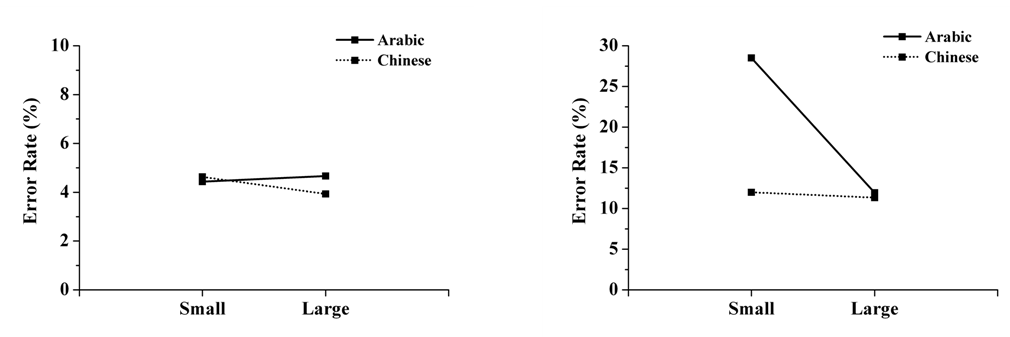
**

**
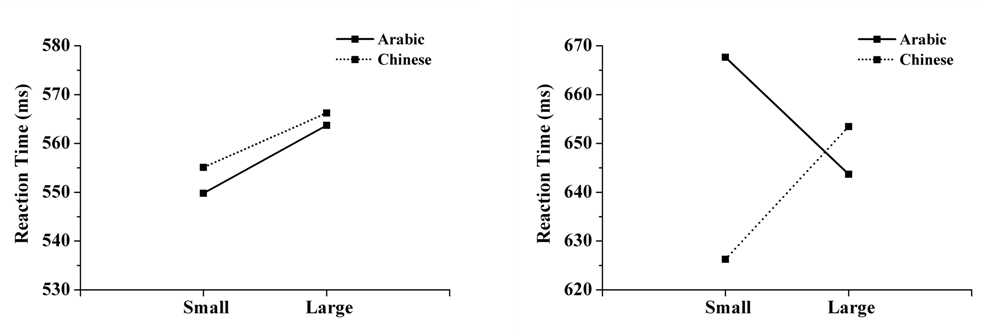
**

**Fig.4** Number Notation effect of Small and Large numerals in the attended condition without cue

**Fig.5** Number Notation effect of Small and Large numerals in the unattended condition without cue. Only small numerals showed significant notation effect

1.2) Parity judgment without cue


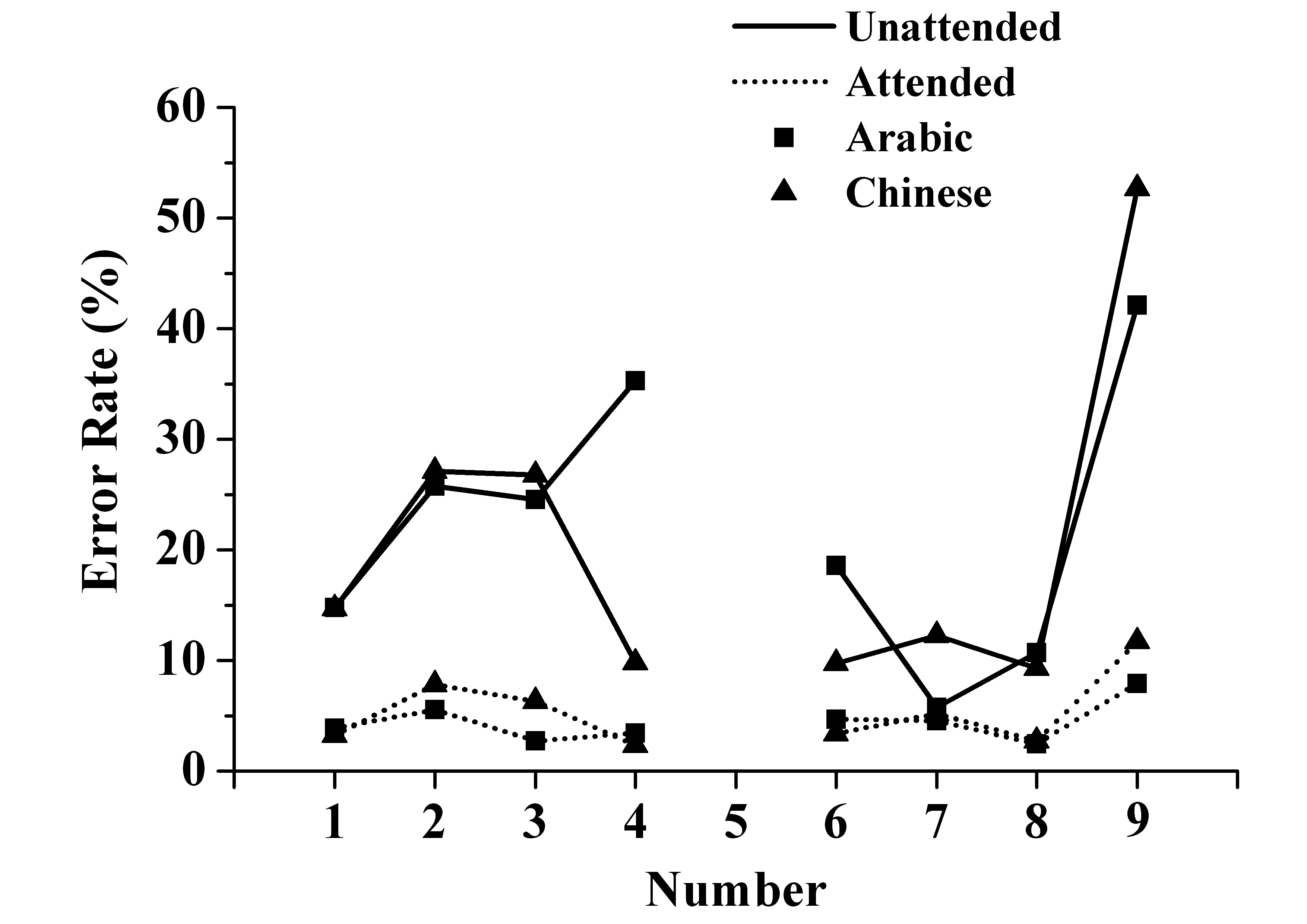

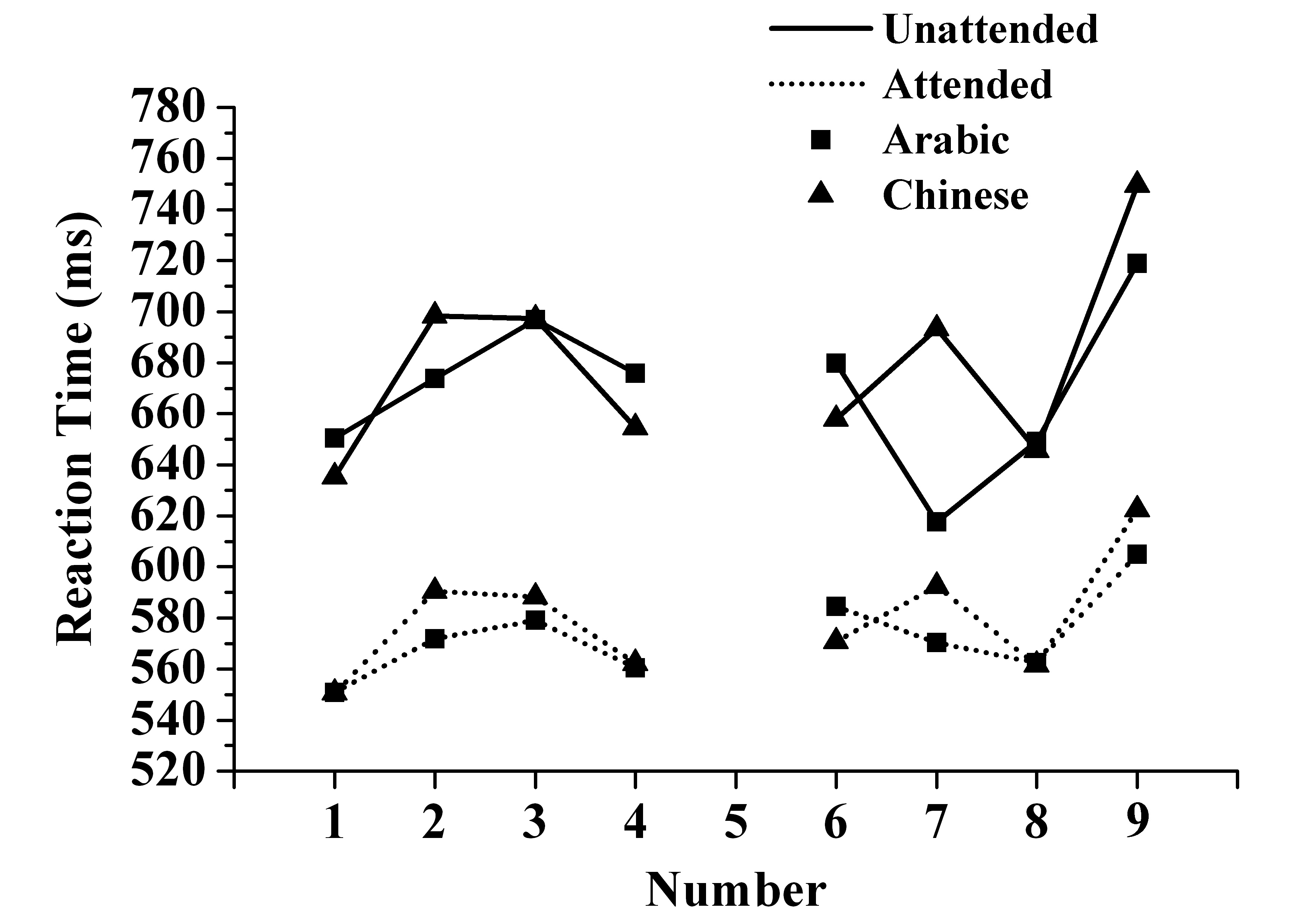


**Fig.6** Error rates of parity judgment condition without cue. No difference between small Chinese and Arabic numerals was found

**Fig.7** RTs of parity judgment without cue. No difference between small Chinese and Arabic numerals was found


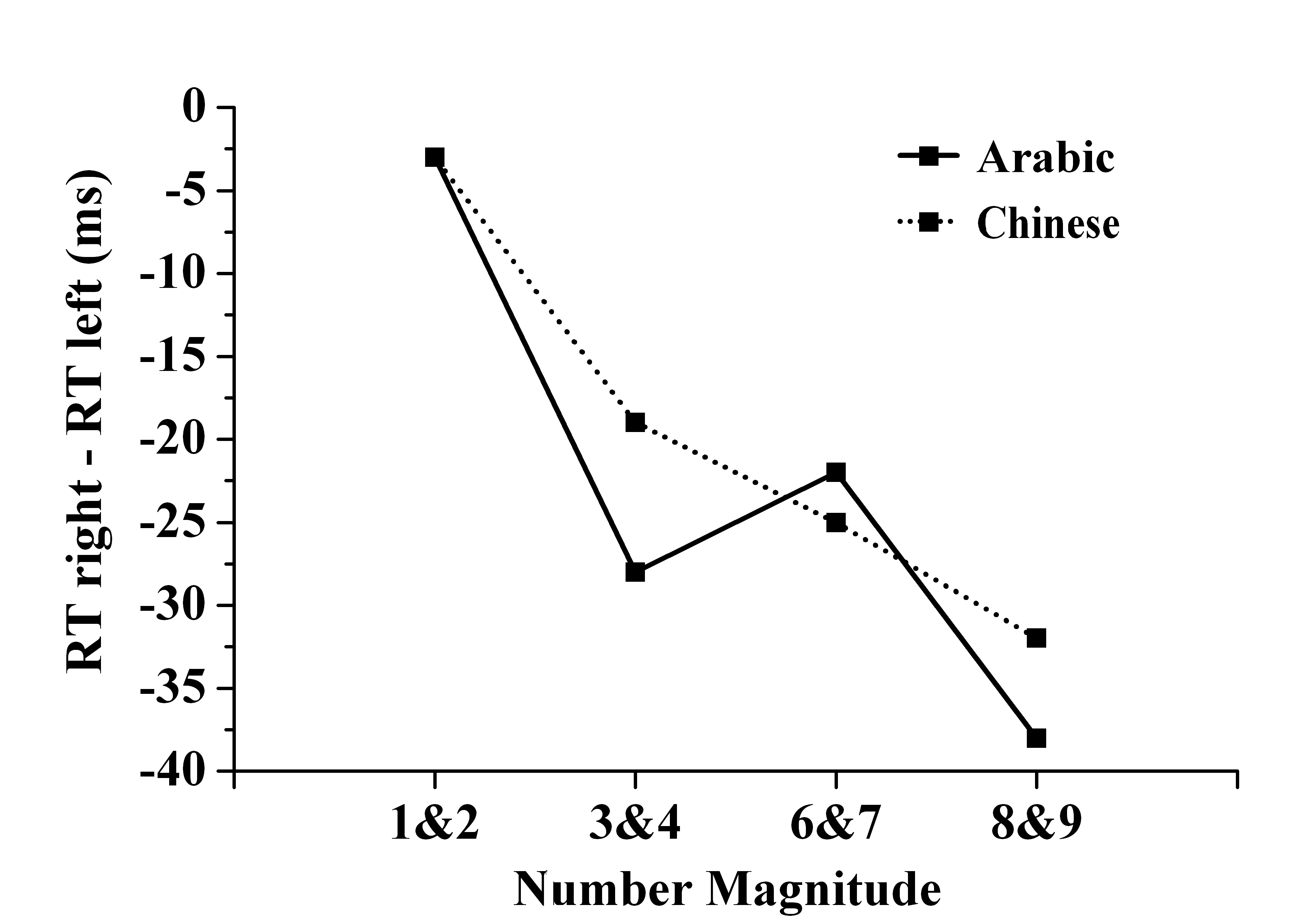

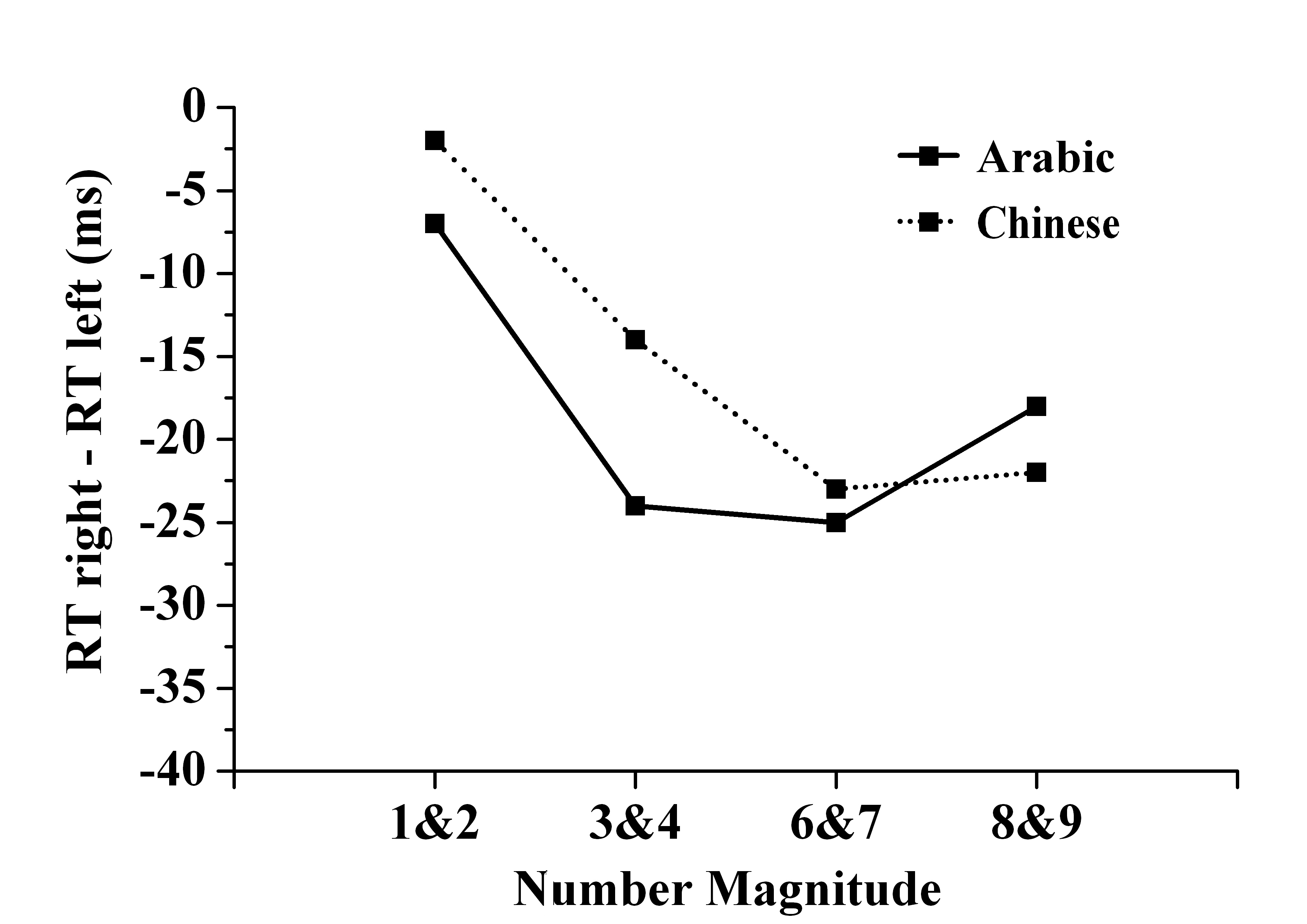


**Fig.8** SNARC effect of Chinese and Arabic numbers in the attended condition without cue

**Fig.9** SNARC effect of Chinese and Arabic numerals in the unattended condition without cue. The SNARC effect was attenuated for large numerals 8&9 in both notations, indicates the influence of attention on the left-to-right internal number line, such that the lack of attention resources for large numerals in the unattended condition omitted the SNARC effect.

**2. Study two: Number processing in different attention conditions with exogenous cue**


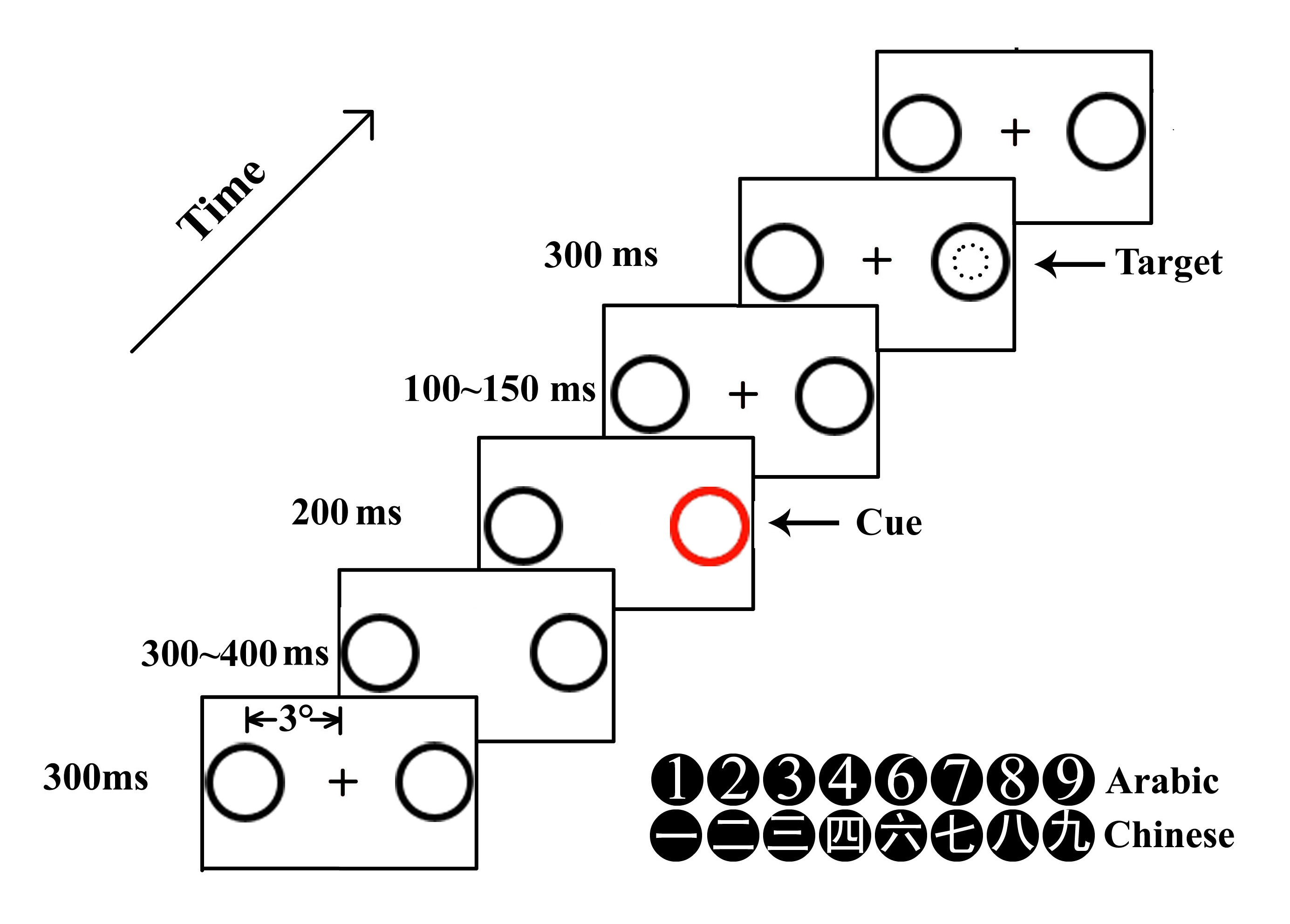


**Fig.10** Procedure (a) A black cross (0.4°) appeared as fixation in the center for 300 ms; (b) A background screen appeared for an interval of 300~400 ms; (c) One of the two background rings turns into a red ring as a target location cue for 200ms; (d) A background screen appeared for an interval of 100~150 ms; (e) One of the sixteen numerals appeared randomly in one of two rings (75% corresponded to the cue (attended),25% did not (unattended)) for 300 ms; (d) A background screen appeared until the participant responded. Participants could respond as soon as the numeral appeared. There was a 500 ms interval before the next trial.

2.1) Magnitude comparison (to 5) with exogenous cue


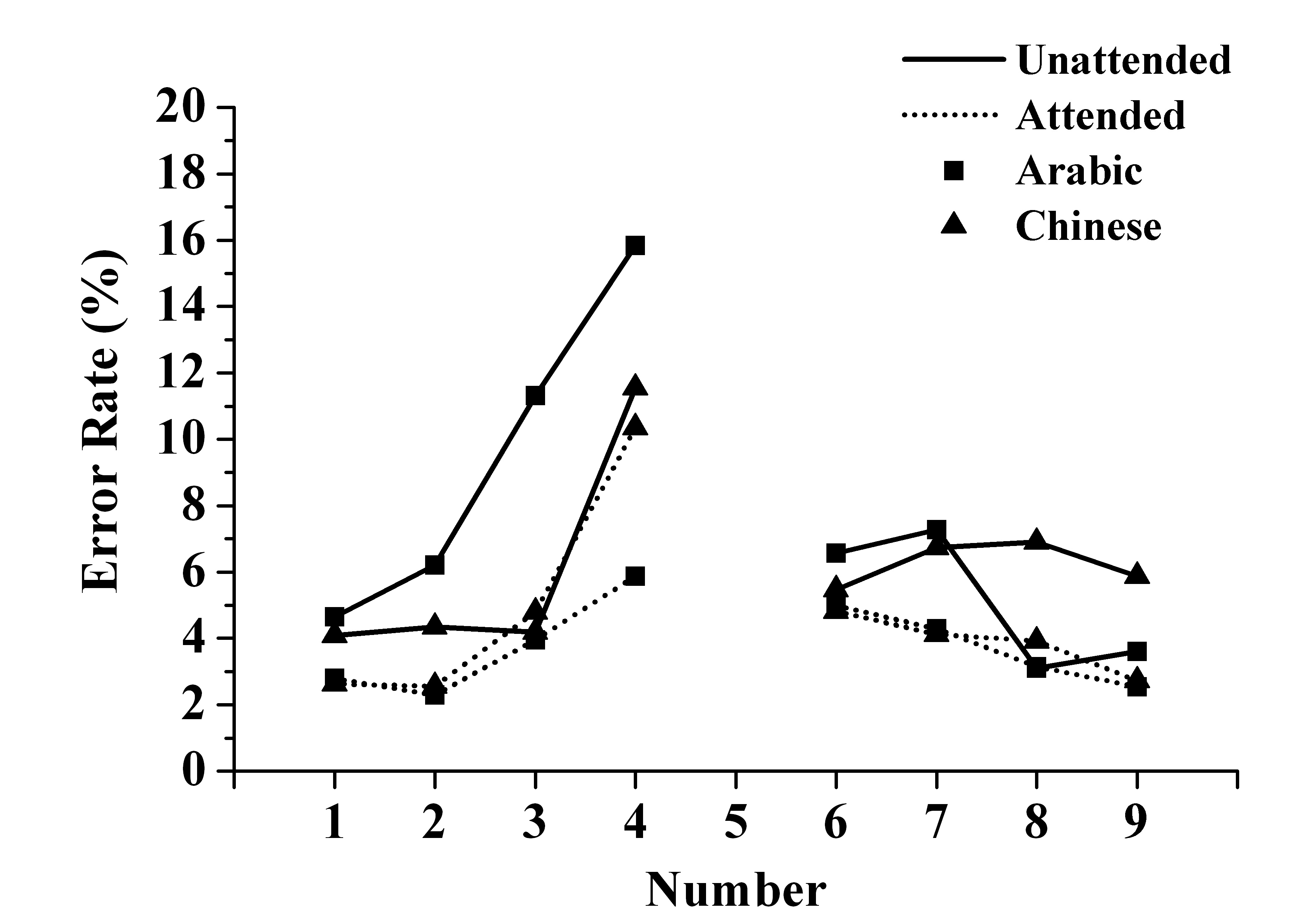

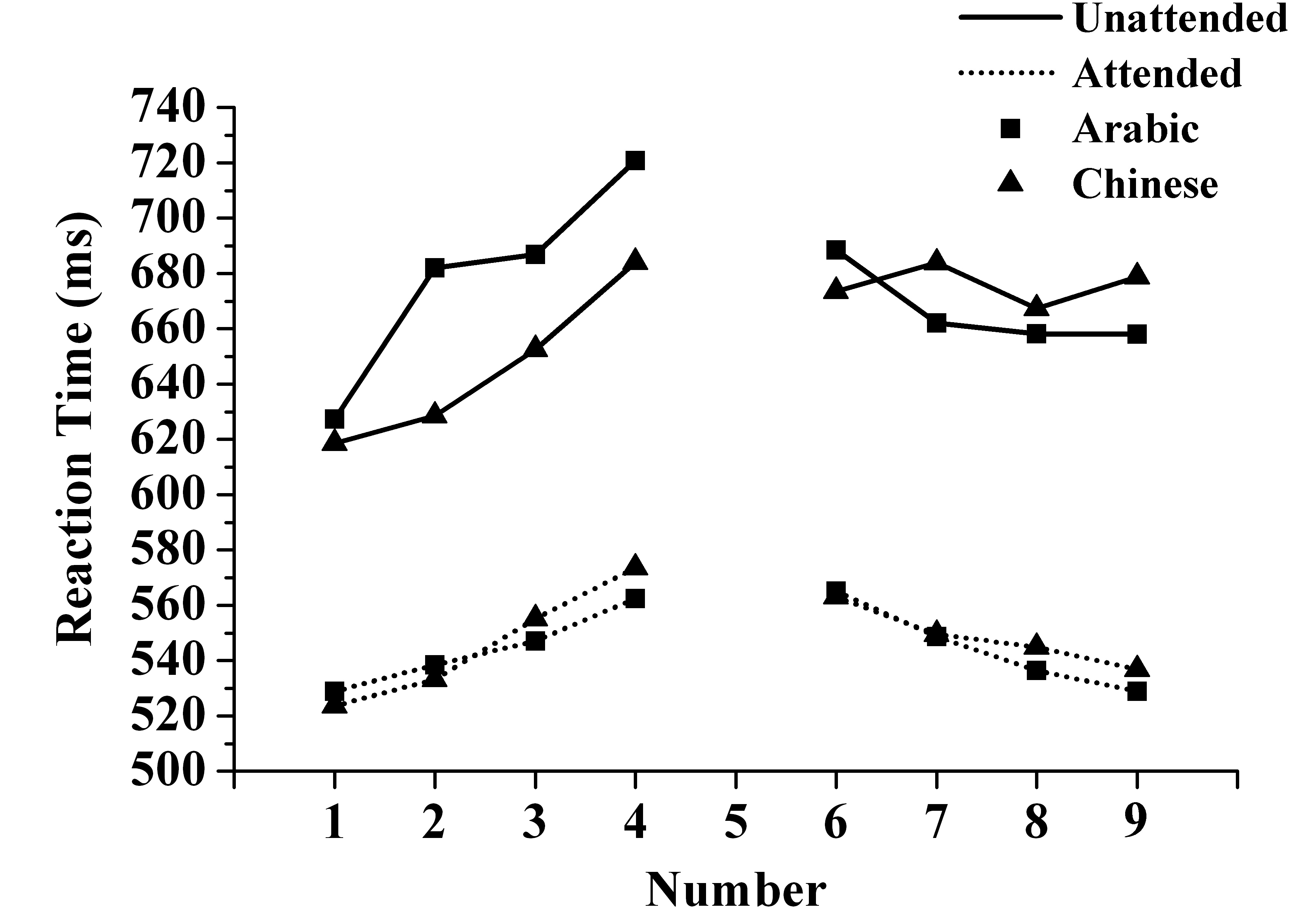


**Fig.11** Error rates of Magnitude comparison with exogenous cue

**Fig.12** RTs of Magnitude comparison with exogenous cue

**
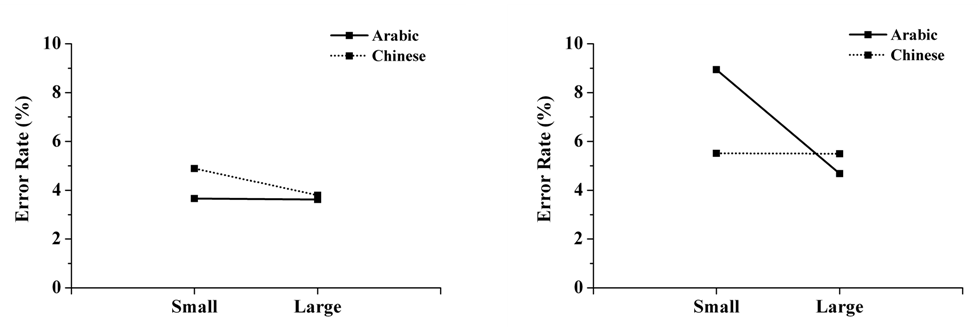
**

**
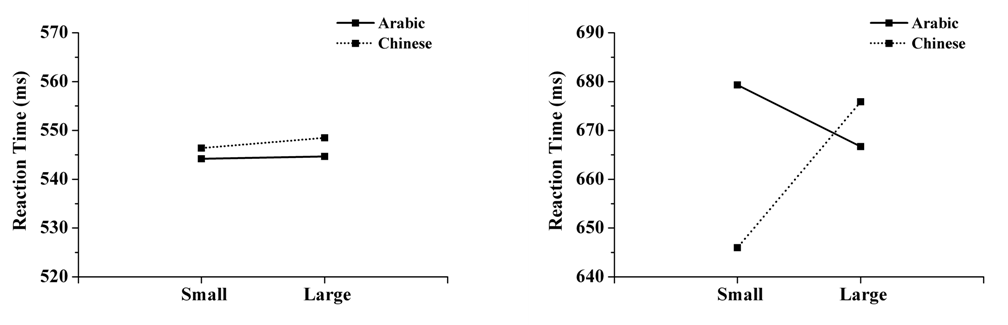
**

**Fig.13** Number Notation effect of Small and Large numerals in the attended condition with exogenous cue

**Fig.14** Number Notation effect of Small and Large numerals in the unattended condition without cue. Only small numerals showed significant notation effect

2.2) Parity judgment with exogenous cue


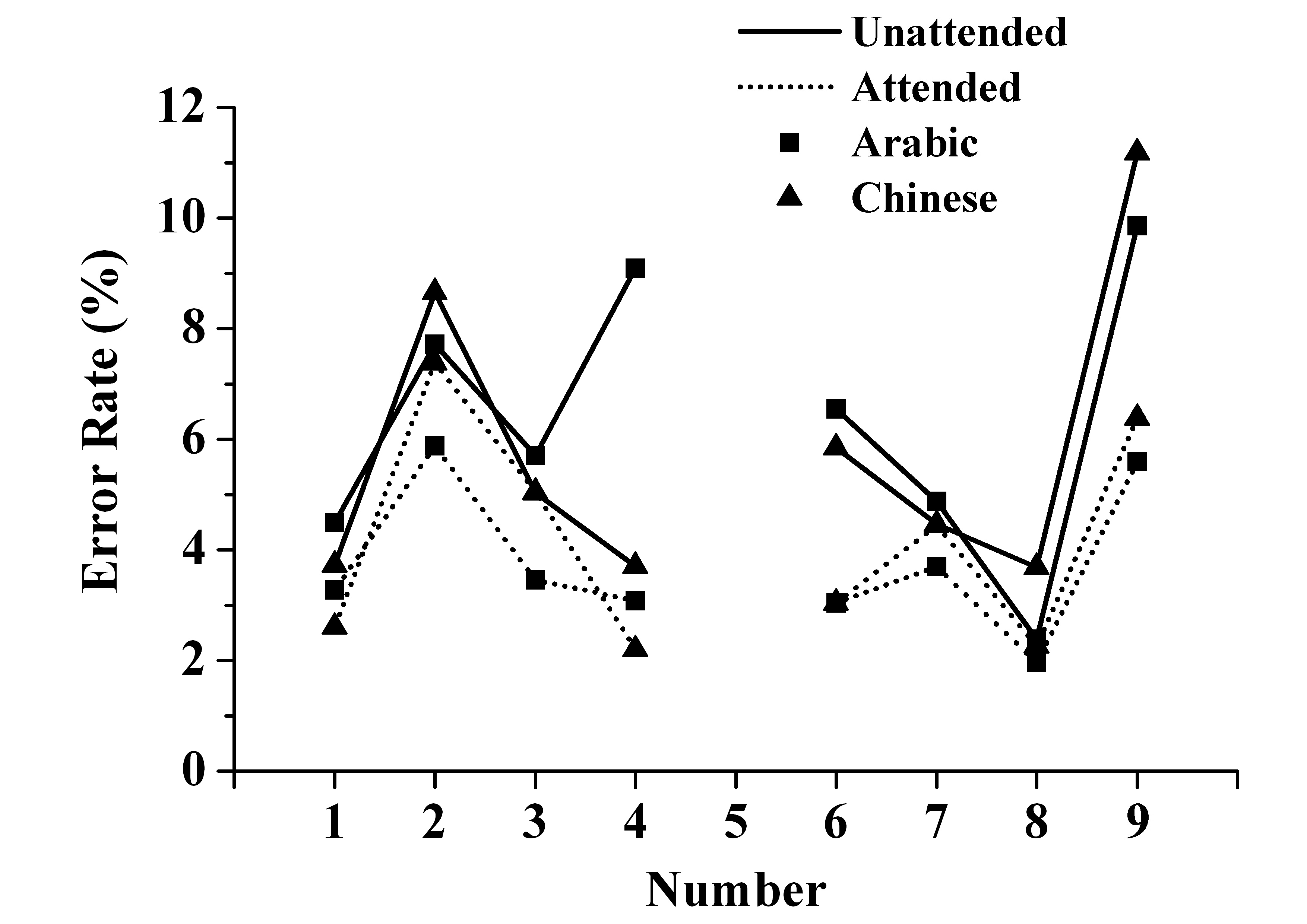

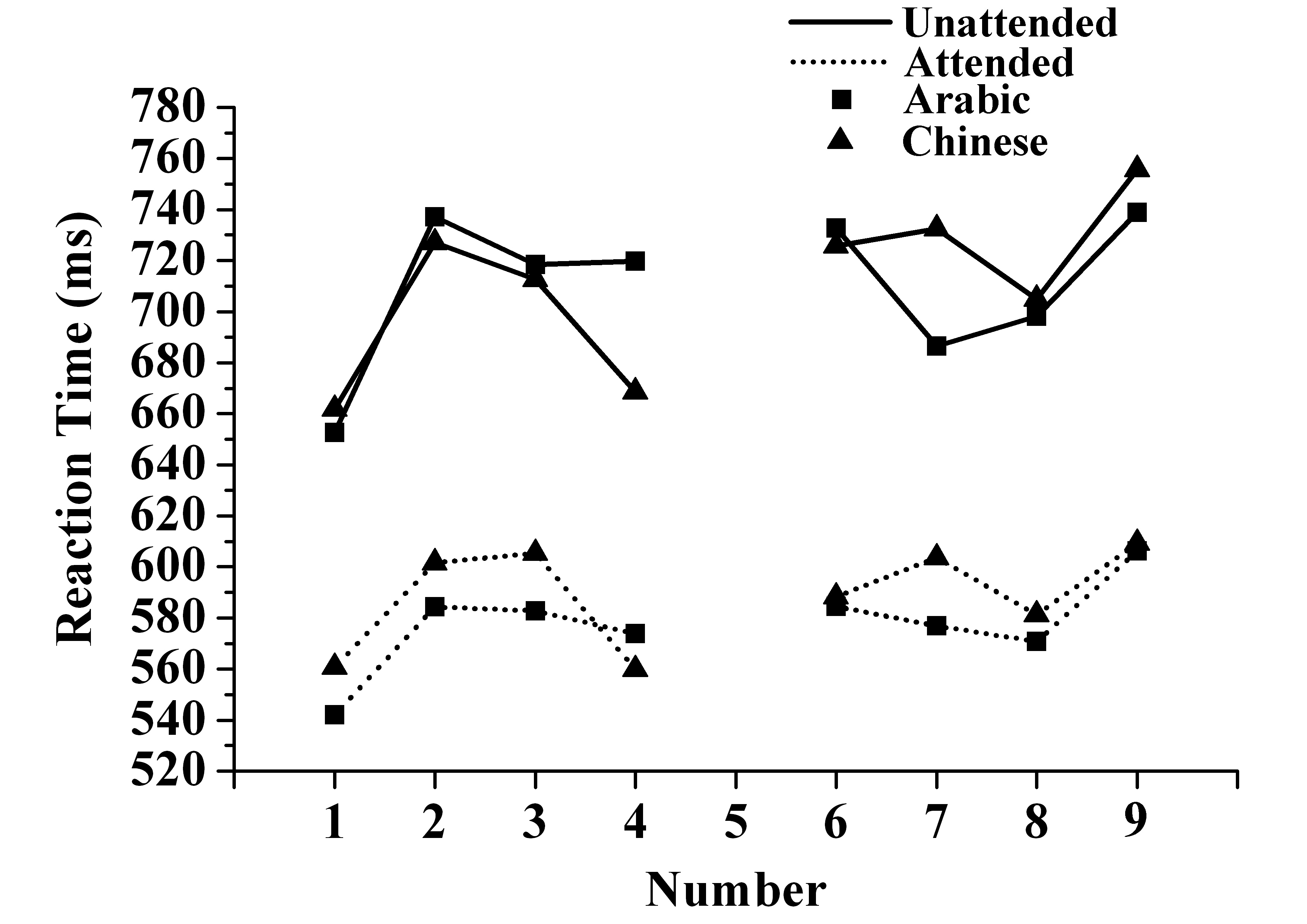


**Fig.15** Error rates of Parity judgment with exogenous cue. No difference between small Chinese and Arabic numerals was found

**Fig.16** Error rates of Parity judgment with exogenous cue. No difference between small Chinese and Arabic numerals was found


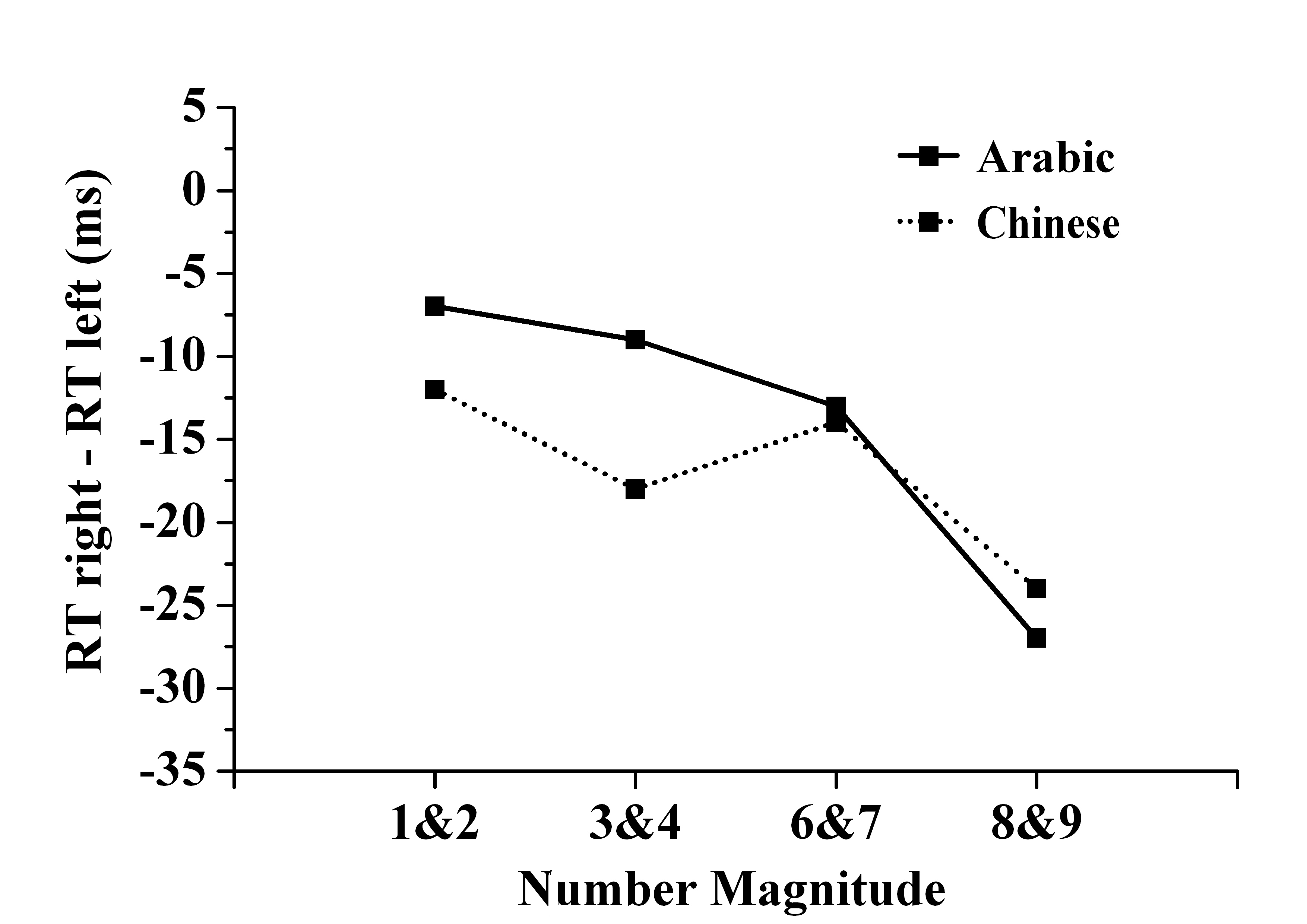

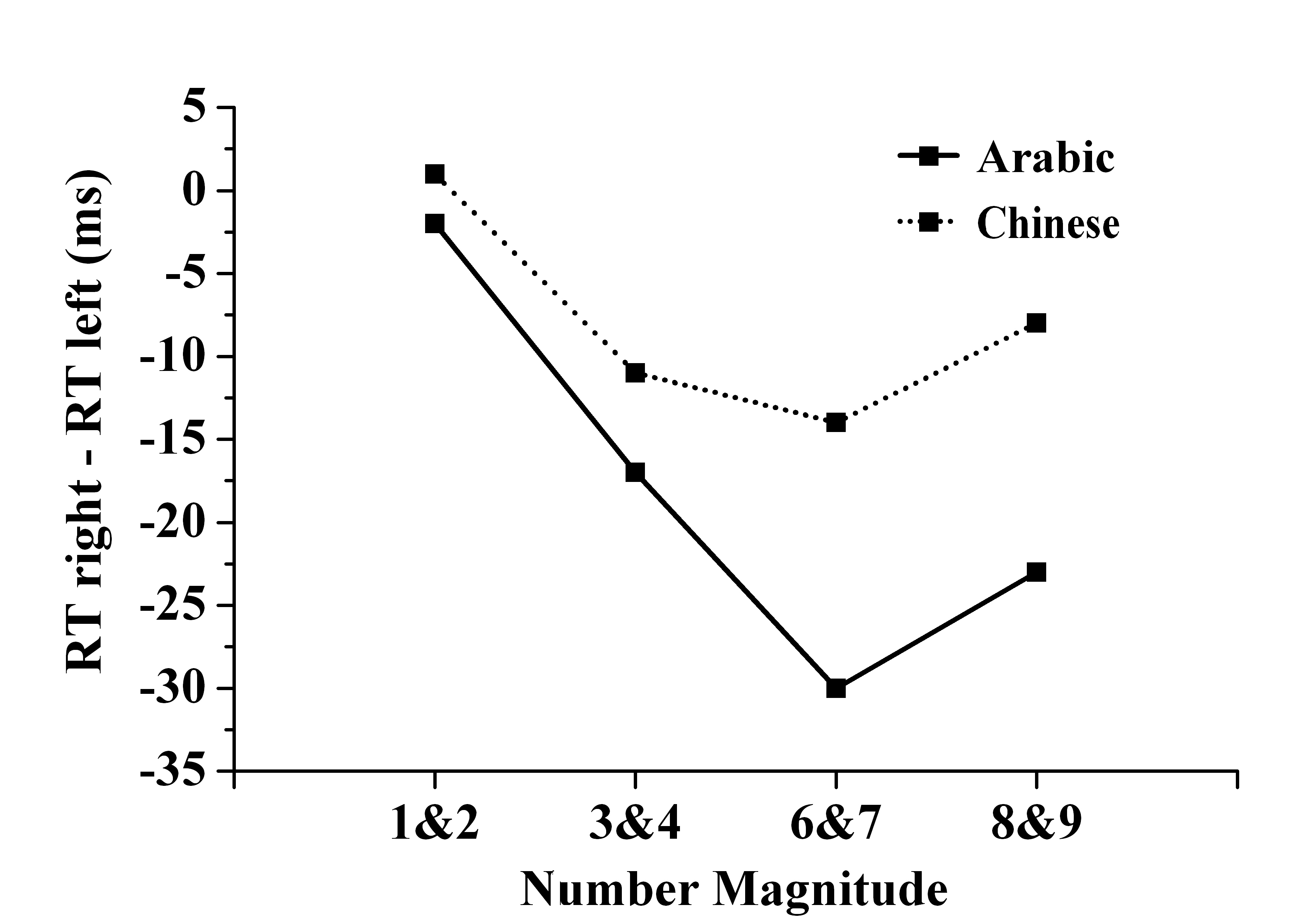


**Fig.17** SNARC effect of Chinese and Arabic numerals in the attended condition with exogenous cue

**Fig.18** SNARC effect of Chinese and Arabic numerals in the unattended condition with exogenous cue. The SNARC effect was attenuated for large numeral 8&9 in both notations, indicates the influence of attention on the left-to-right internal number line, such that the lack of attention resources for large numerals in the unattended condition omitted the SNARC effect.

## 3. Study three: Number processing in different attention conditions with endogenous cue


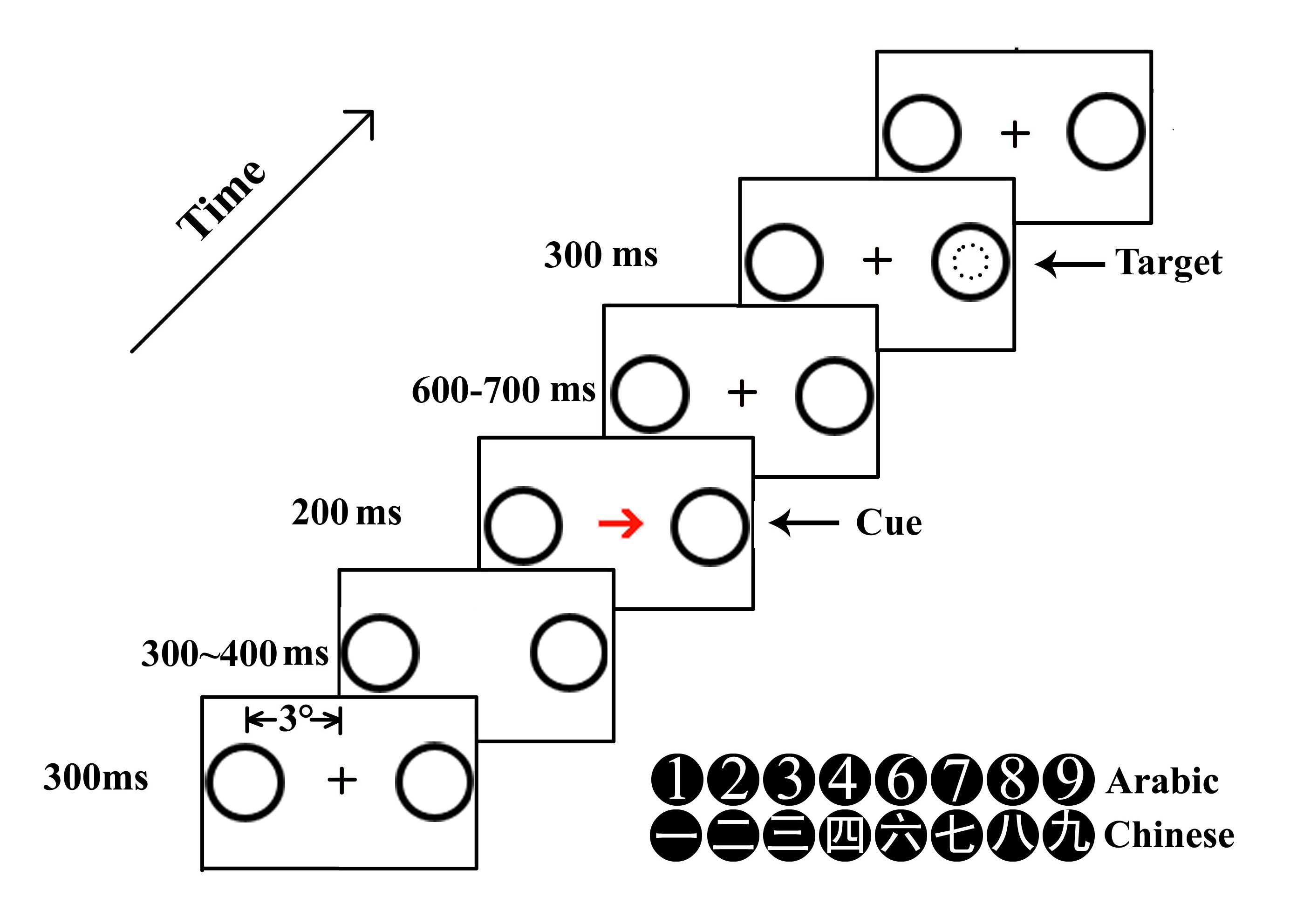


**Fig.19** Procedure (a) A black cross (0.4°) appeared as fixation in the center for 300 ms; (b) A background screen appeared for an interval of 300~400 ms; (c) A red arrow (0.4 *0.2) appeared as a target location cue for 200ms; (d) A background screen appeared for an interval of 600~700 ms; (e) One of the sixteen numerals appeared randomly in one of two rings (75% corresponded to the cue (attended), 25% did not(unattended)) for 300 ms; (d) A background screen appeared until the participant responded. Participants could respond as soon as the number appeared. There was a 500 ms interval before the next trial.

3.1) Magnitude comparison (to 5) with endogenous cue


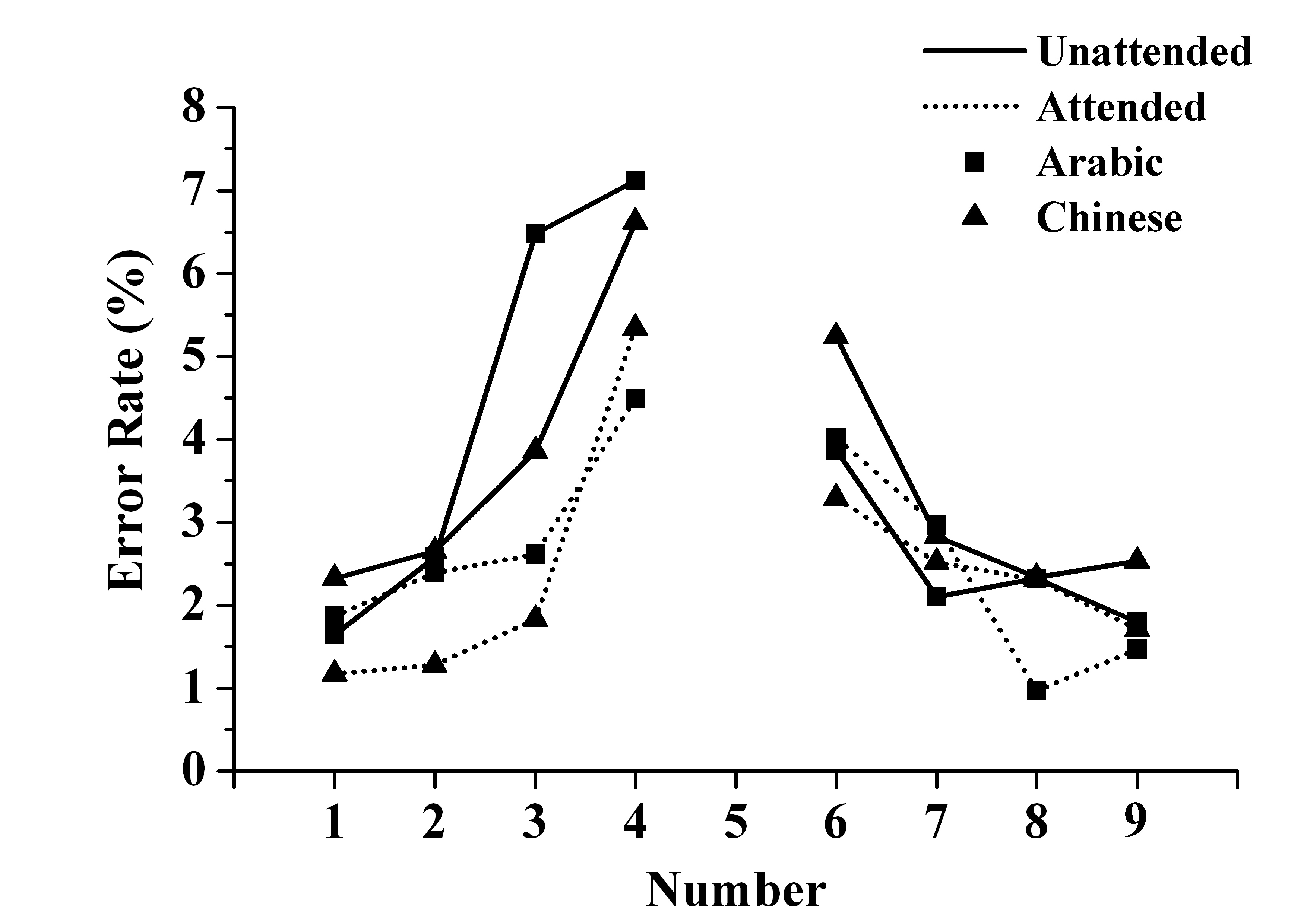

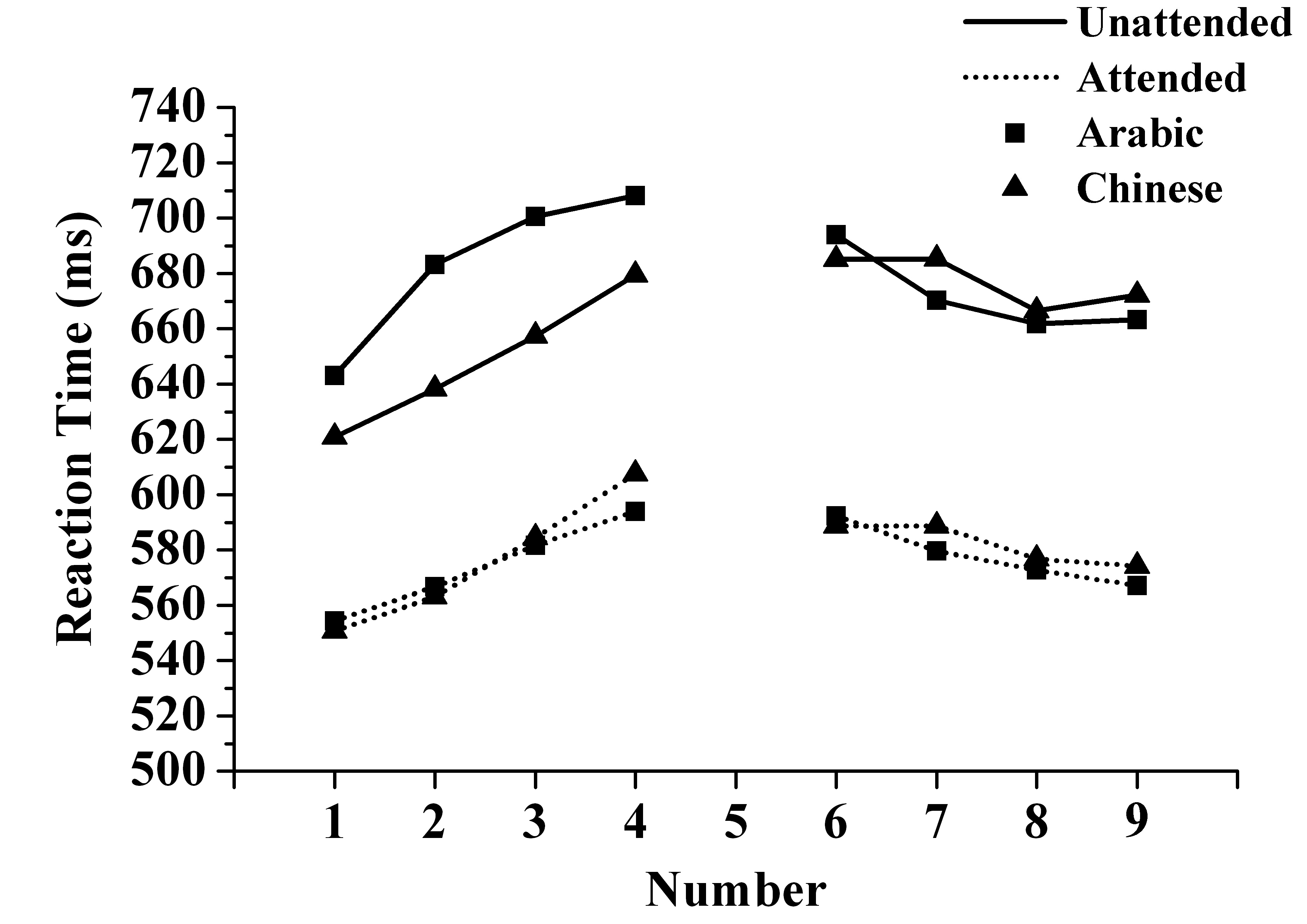


**Fig.20** Error rates of Magnitude comparison with endogenous cue

**Fig.21** RTs of Magnitude comparison with endogenous cue

**
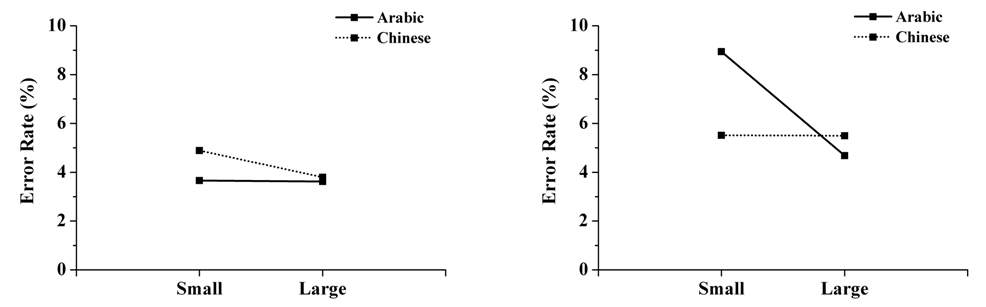
**

**
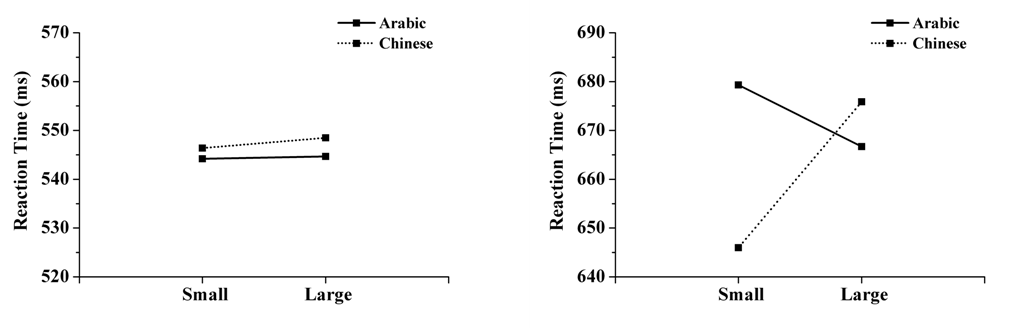
**

**Fig.22** Number Notation effect of Small and Large numerals in the attended condition with endogenous cue

**Fig.23** Number Notation effect of Small and Large numerals in the unattended condition with endogenous cue. Only small numerals showed significant notation effect

3.2) Parity judgment with endogenous cue


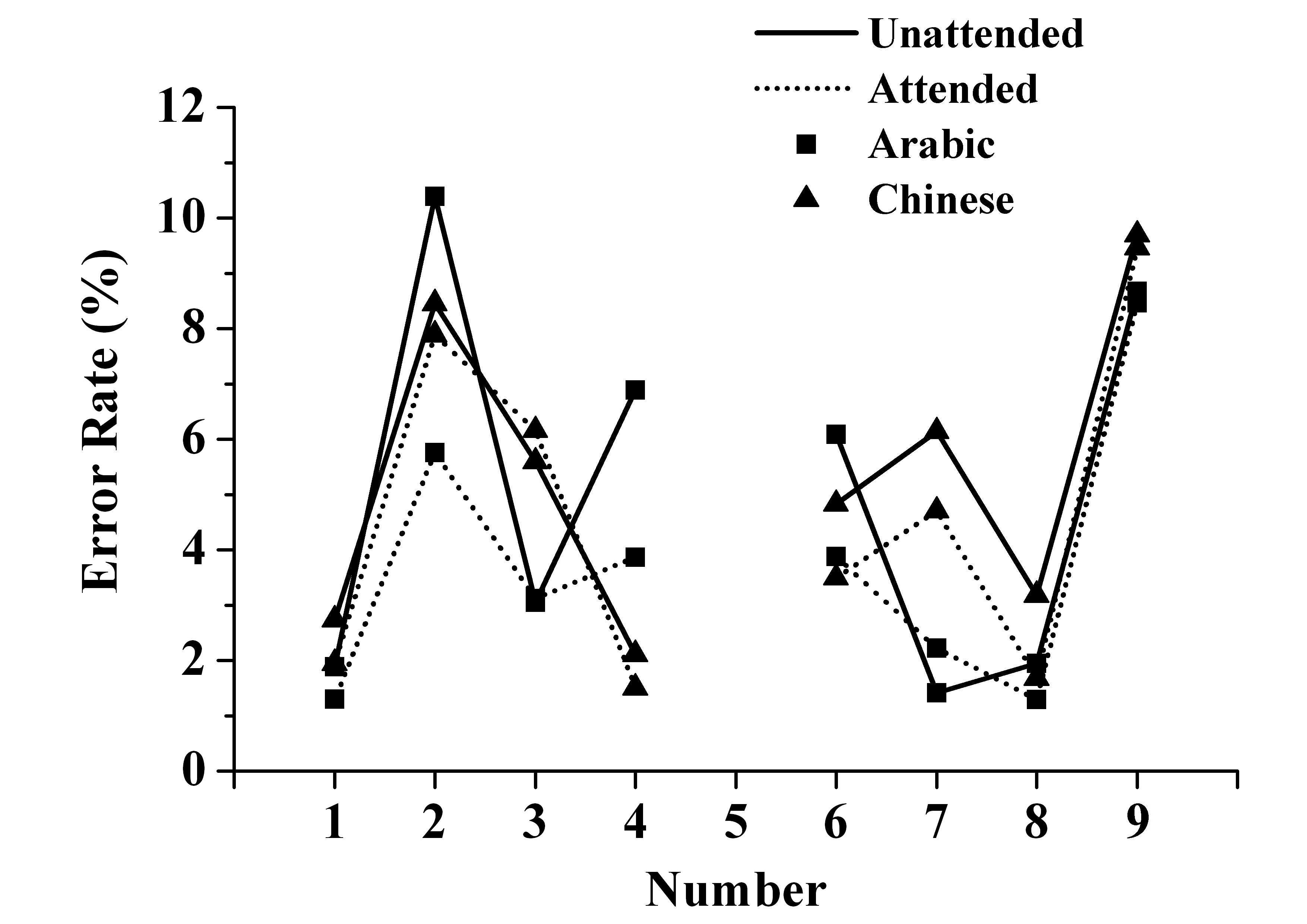

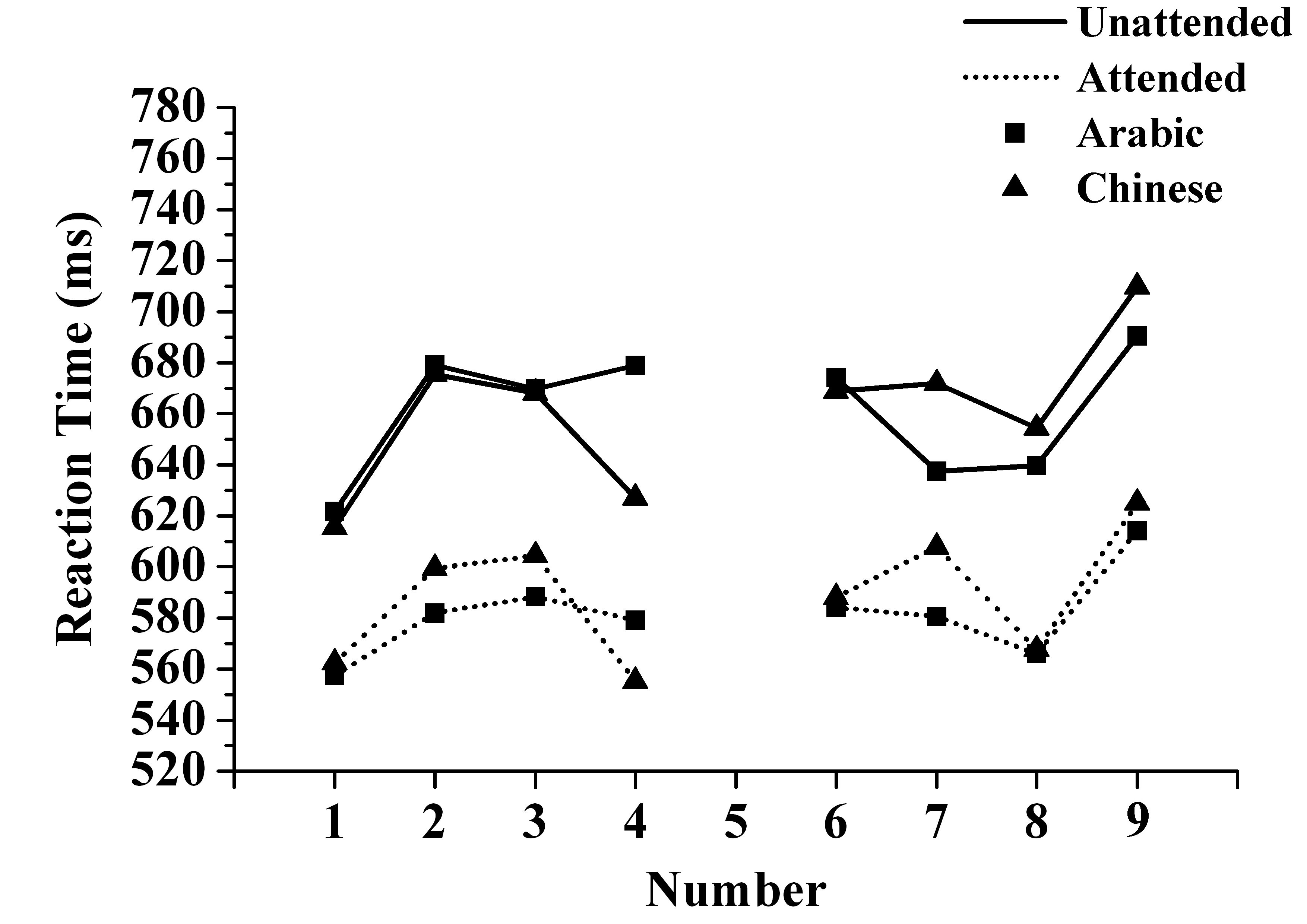


**Fig.24** Error rates of Parity judgment with endogenous cue. No difference between small Chinese and Arabic numerals was found

**Fig.25** RTs of Parity judgment with endogenous cue. No difference between small Chinese and Arabic numerals was found


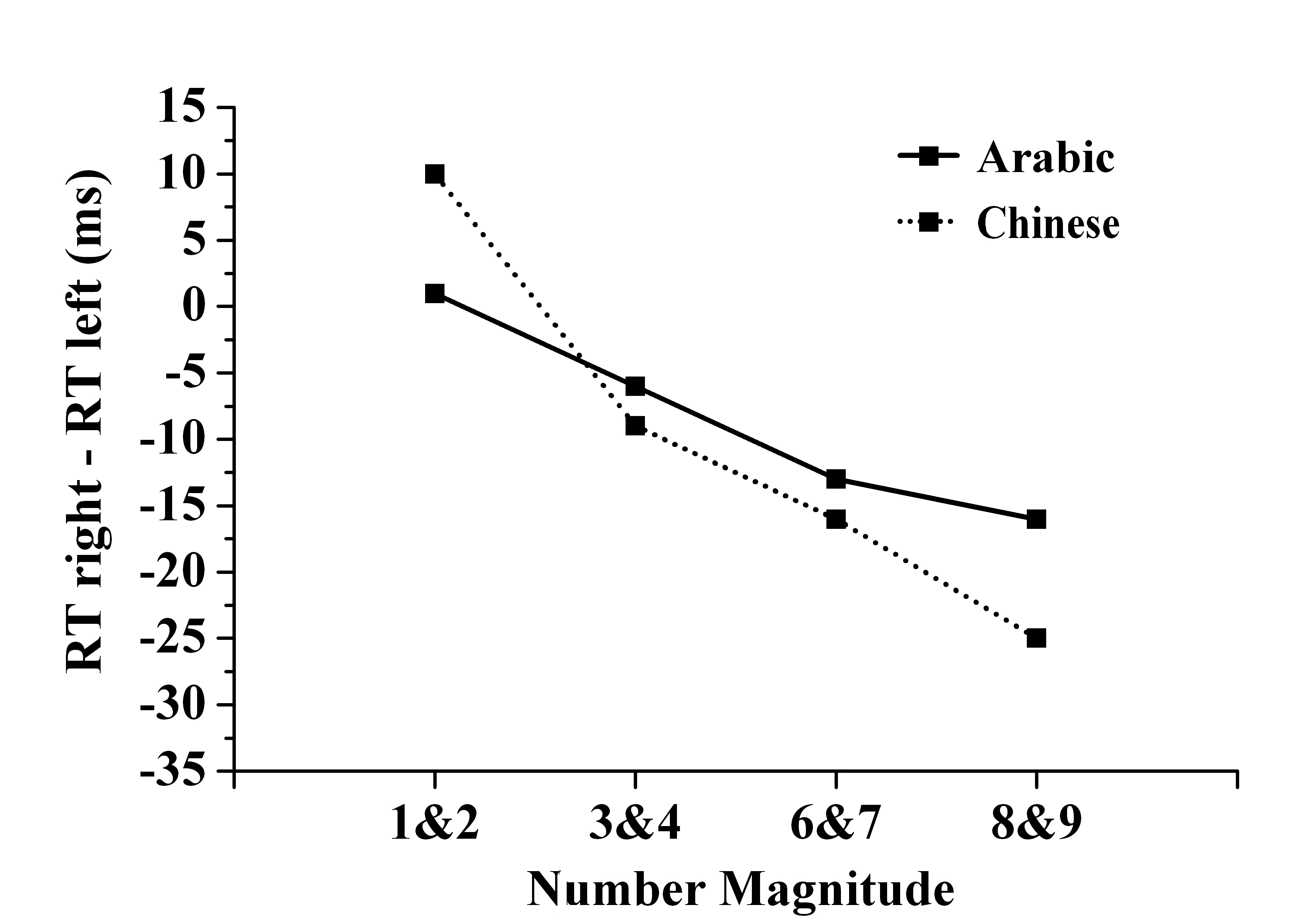

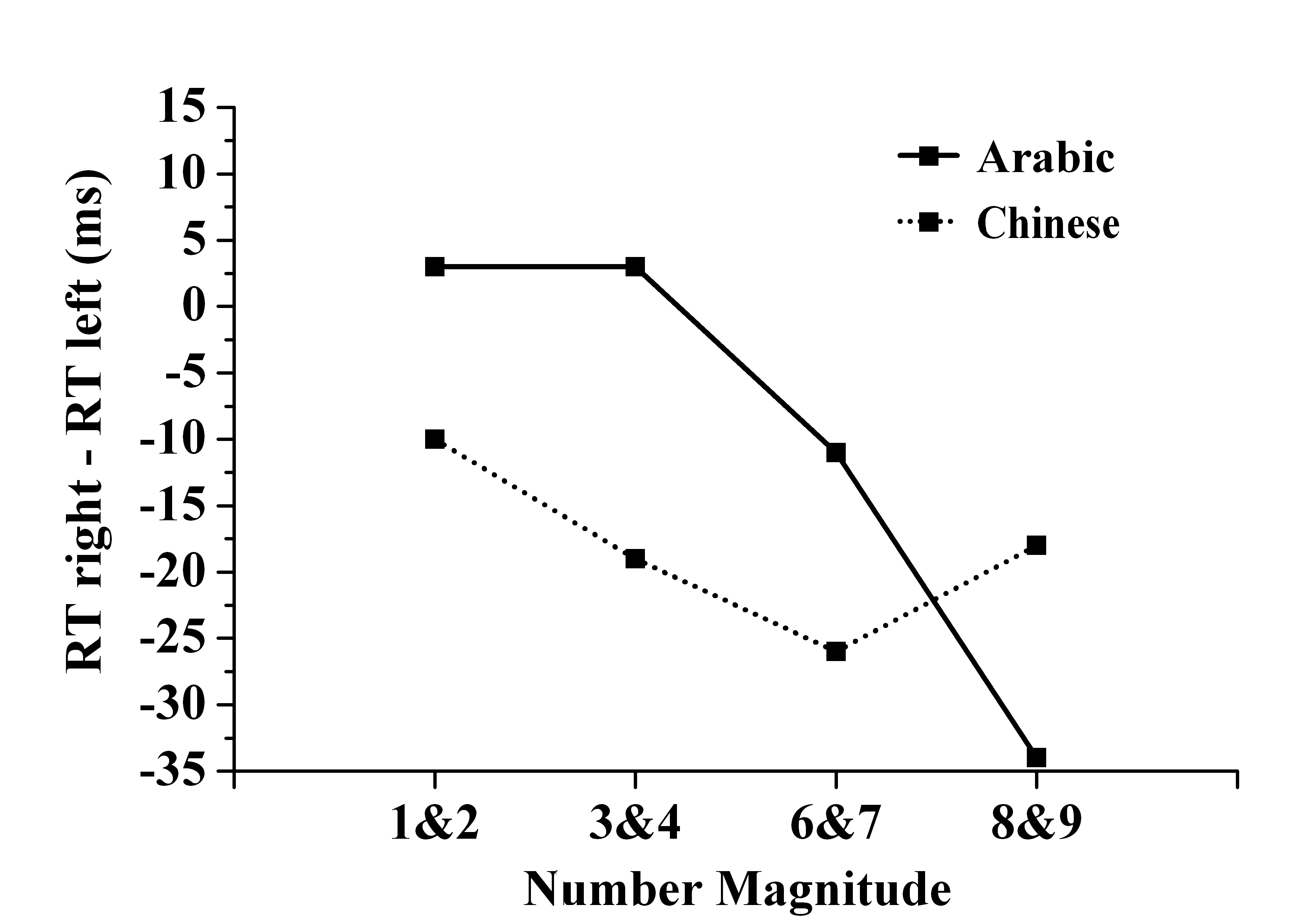


**Fig.26** SNARC effect of Chinese and Arabic numerals in the attended condition with endogenous cue

**Fig.27** SNARC effect of Chinese and Arabic numerals in the unattended condition with endogenous cue. The SNARC effect was attenuated for large numeral 8&9 only in Chinese numerals, indicates endogenous attention influences the left-to-right internal number line differently for Chinese and Arabic numerals.
